# Supplementary material for: Coordinated hippocampal-thalamic-cortical communication crucial for engram dynamics underneath systems consolidation
Source: Nat Commun. 2022 Feb 11;13:840. doi: 10.1038/s41467-022-28339-z (PMC8837777; doi:10.1038/s41467-022-28339-z)
Supplement: Supplementary file 1 — Supplementary Information [file 41467_2022_28339_MOESM1_ESM.pdf]

Supplementary Information

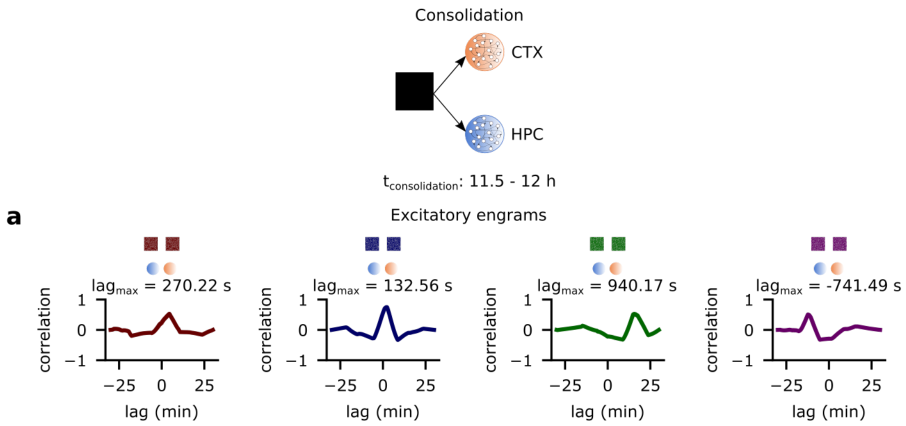

Supplementary Figure 1. Decoupled engram reactivations in circuit with disconnected HPC and CTX. a, Cross-correlograms of the population activities of excitatory engram cells encoding the same stimulus in HPC and CTX during the consolidation phase of Fig. 1b (see Methods for the definition of lag_{max}). Pair of stimuli indicated at the top. Pair of regions indicated at the top of each cross-correlogram. Time interval: between 11.5 and 12 h of consolidation. Color and stimuli as in Fig. 1a.

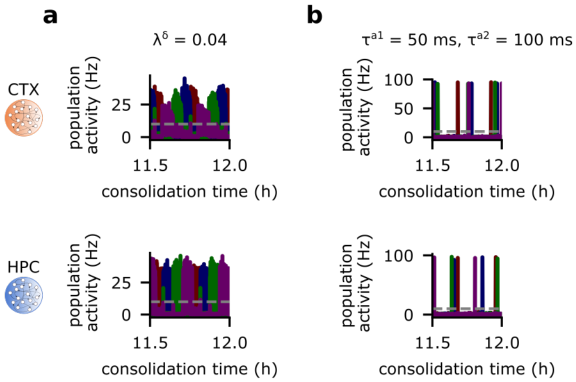

Supplementary Figure 2. Transmitter-induced plasticity strength and neuronal adaptation time constants shape engram reactivations. **a**, Population activity of engram cells in the consolidation phase of Fig. 1b when $\lambda^{\delta} = 0.04$ (see Methods). All other simulation and network parameters as in Fig. 1a. Each color designates the engram cells encoding the respective stimulus in Fig. 1a. Dashed line indicates threshold $\zeta^{thr} = 10$ Hz for engram cell activation. Top: CTX. Bottom: HPC. **b**, Population activity of engram cells in the consolidation phase of Fig. 1b when neurons are subject to spike-triggered adaptation with two time constants: $\tau^{a1} = 50$ ms and $\tau^{a2} = 100$ ms (see Methods). All other simulation and network parameters as in Fig. 1a. Each color designates the engram cells encoding the respective stimulus in Fig. 1a. Dashed line indicates threshold $\zeta^{thr} = 10$ Hz for engram cell activation. Top: CTX. Bottom: HPC. **a-b**, Color as in Fig. 1a.

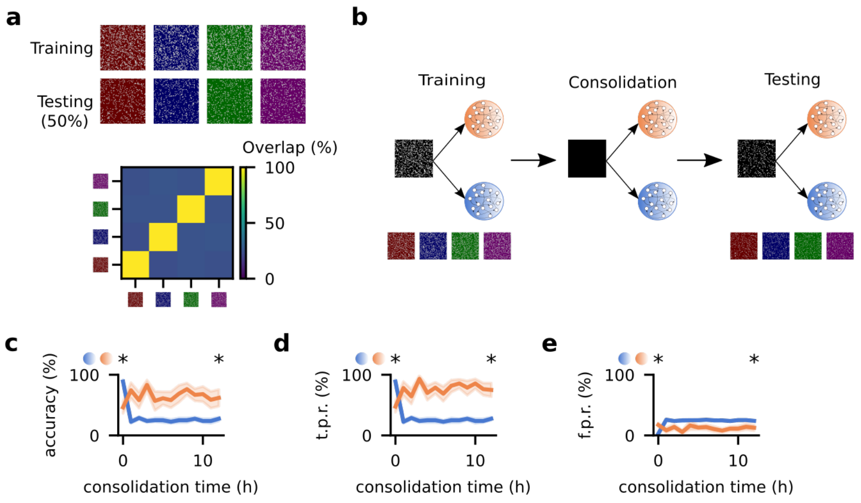

Supplementary Figure 3. Engrams encoding overlapping stimuli are subject to maturation in CTX and de-maturation in HPC. a, Top: Overlapping stimuli presented in the training phase with their respective partial cues used in the testing phase. Light gray indicates active neurons firing above baseline whereas the remaining background color (i.e., red/blue/green/purple) indicates neurons at baseline firing rate. Each background color denotes a distinct stimulus and its respective partial cue. Each stimulus consists of a random 25% of the total neurons in the stimulus population. Each partial cue consists of a random 50% of the full stimulus. Bottom: Overlap between each pair of stimuli. Average overlap between two different stimuli is 25%. **b**, Schematic of simulation protocol with overlapping stimuli. Network configuration and simulation parameters as in Fig. 1a-b except that training time $T_{training} = 55$ min. **c-e**, Memory recall in the testing phase of protocol **b** as a function of consolidation time. $n = 10$ trials. Mean values and 90% confidence intervals shown. **c**, Recall accuracy. Two-sided Mann-Whitney U test between accuracy in HPC and CTX at consolidation time = 0 (p-value $= 2.465092 \cdot 10^{-4}$) and 12 h (p-value $= 1.565696 \cdot 10^{-3}$). **d**, Recall true positive rate. Two-sided Mann-Whitney U test between true positive rate in HPC and CTX at consolidation time = 0 (p-value $= 2.451567 \cdot 10^{-4}$) and 12 h (p-value $= 3.044753 \cdot 10^{-4}$). **e**, Recall false positive rate. Two-sided Mann-Whitney U test between false positive rate in HPC and CTX at consolidation time = 0 (p-value $= 1.503205 \cdot 10^{-4}$) and 12 h (p-value $= 1.587860 \cdot 10^{-3}$). *p-value < 0.05 (see Methods). **b-e**, Color as in Fig. 1a.

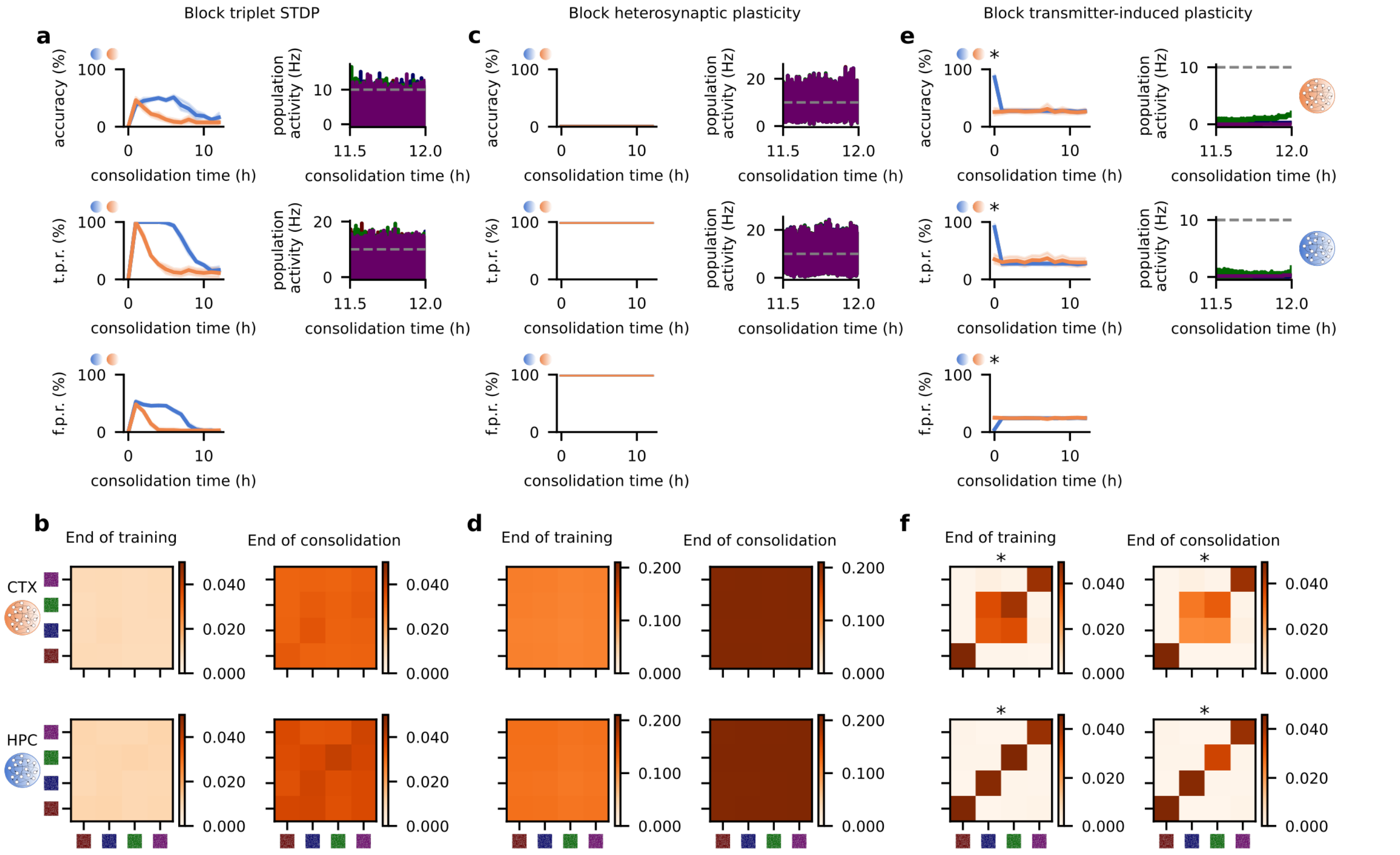

Supplementary Figure 4. Hebbian and non-Hebbian forms of synaptic plasticity are essential for consistent engram dynamics. a, Analysis of memory recall and population activity of the network in Fig. 1a when the triplet STDP component of synaptic plasticity is blocked throughout the protocol in Fig. 1b. Left: Memory recall accuracy (top), true positive rate (middle), and false positive rate (bottom) as a function of consolidation time. Mean values and 90% confidence intervals shown. For each recall metric, two-sided Mann-Whitney U test. Accuracy: consolidation time = 0 (HPC vs. CTX p-value = 0.920340) and 12 h (HPC vs. CTX p-value = 0.200256). True positive rate: consolidation time = 0 (HPC vs. CTX p-value = 0.920340) and 12 h (HPC vs. CTX p-value = 0.333666). False positive rate: consolidation time = 0 (HPC vs. CTX p-value = 0.920340) and 12 h (HPC vs. CTX p-value = 0.366053). Right: Population activity of engram cells encoding each stimulus in CTX (top) and HPC (bottom) (each color designates the engram cells encoding the respective stimulus in Fig. 1a; dashed line indicates threshold $\zeta^{thr} = 10$ Hz for engram cell activation). **b**, Mean weight strength of recurrent excitatory synapses onto excitatory neurons at the end of the training phase (left) and after 12 hours of consolidation (right) clustered according to engram cell preference. Top: CTX. Bottom: HPC. Two-sided Kolmogorov-Smirnov test between the distribution of recurrent excitatory weights among engram cells encoding the same stimulus (i.e., diagonal) and that of recurrent excitatory weights among engram cells encoding different stimuli (i.e., off-diagonal) at the end of training (CTX: p-value = 0.702617; HPC: p-value = 0.472777) and after 12 of consolidation (CTX: p-value = 0.709640; HPC: p-value = 0.489169). **c-d**, Same as **a-b** but blocking heterosynaptic plasticity. Recall curves: mean values and 90% confidence intervals shown. For each recall metric, two-sided Mann-Whitney U test. Accuracy: consolidation time = 0 (HPC vs. CTX p-value = 0.920340) and 12 h (HPC vs. CTX p-value = 0.920340). True positive rate: consolidation time = 0 (HPC vs. CTX p-value = 0.920340) and 12 h (HPC vs. CTX p-value = 0.920340). False positive rate: consolidation time = 0 (HPC vs. CTX p-value = 0.920340) and 12 h (HPC vs. CTX p-value = 0.920340). Mean weights: two-sided Kolmogorov-Smirnov test between the distribution of recurrent excitatory weights among engram cells encoding the same stimulus (i.e., diagonal) and that of recurrent excitatory weights among engram cells encoding different stimuli (i.e., off-diagonal) at the end of training (CTX: p-value = 1; HPC: p-value = 1) and after 12 of consolidation (CTX: p-value = 1; HPC: p-value = 1). **e-f**, Same as **a-b** but blocking transmitter-induced plasticity. Recall curves: mean values and 90% confidence intervals shown. For each recall metric, two-sided Mann-Whitney U test. Accuracy: consolidation time = 0 (HPC vs. CTX p-value = $1.538463 \cdot 10^{-4}$) and 12 h (HPC vs. CTX p-value = 0.605577). True positive rate: consolidation time = 0 (HPC vs. CTX p-value = $1.823933 \cdot 10^{-4}$) and 12 h (HPC vs. CTX p-value = 0.833964). False positive rate: consolidation time = 0 (HPC vs. CTX p-value = $1.538463 \cdot 10^{-4}$) and 12 h (HPC vs. CTX p-value = 0.605577). Mean weights: two-sided Kolmogorov-Smirnov test between the distribution of recurrent excitatory weights among engram cells encoding the same stimulus (i.e., diagonal) and that of recurrent excitatory weights among engram cells encoding different stimuli (i.e., off-diagonal) at the end of training (CTX: p-value = 0; HPC: p-value = 0) and after 12 of consolidation (CTX: p-value = 0; HPC: p-value = 0). *p-value < 0.05 (see Methods). **a/c**, $n = 5$ trials. **e**, $n = 10$ trials. **a-f**, Network and simulation parameters as in Fig. 1b except that training time $T_{training} = 90$ min in **e-f**. Color and stimuli as in Fig. 1a.

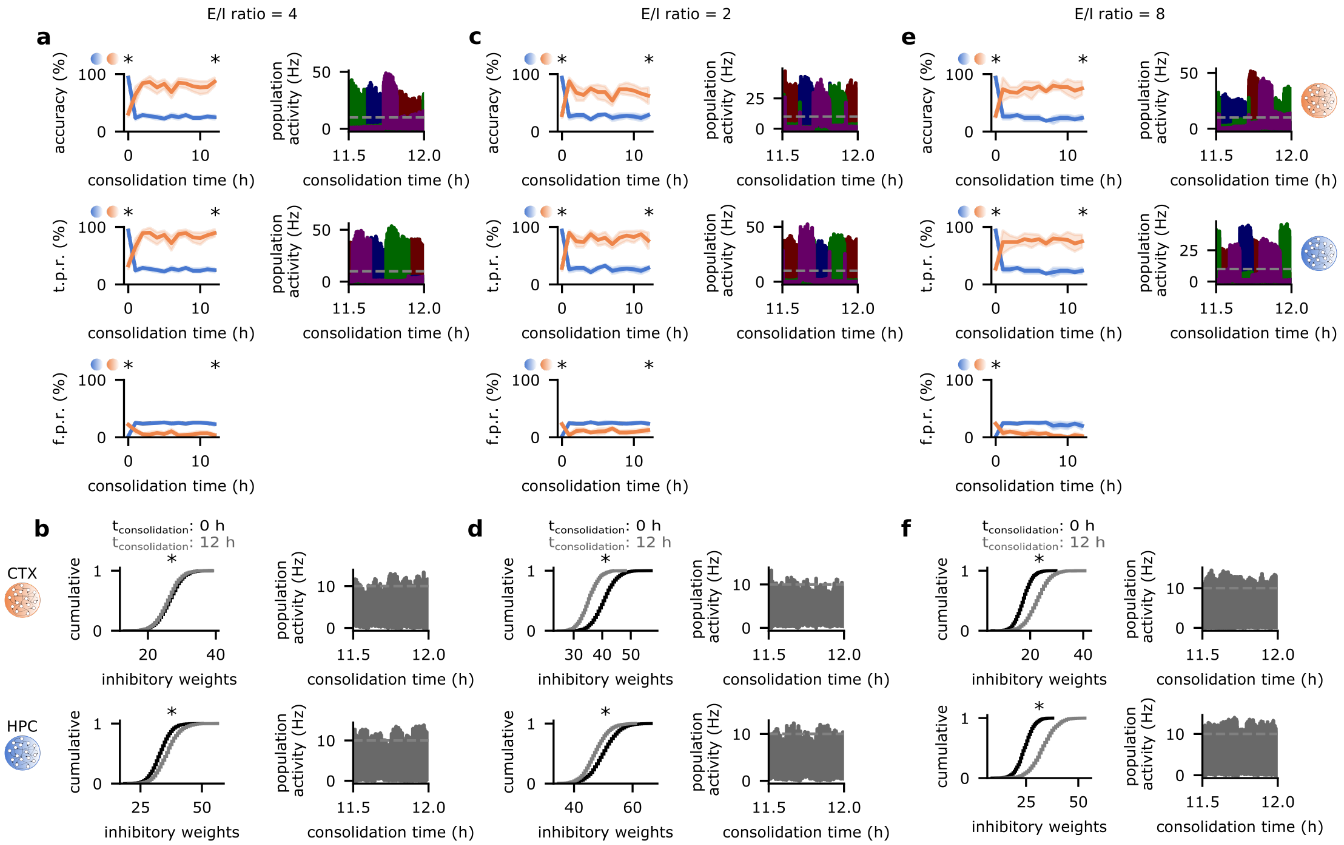

Supplementary Figure 5. Engram dynamics in CTX and HPC are robust to changes in E/I ratio. **a**, Analysis of memory recall and population activity of the network in Fig. 1a subject to the protocol in Fig. 1b with the baseline E/I ratio of 4 for both CTX and HPC (same as Fig. 1c-f). Left: Memory recall accuracy (top), true positive rate (middle), and false positive rate (bottom) as a function of consolidation time. Mean values and 90% confidence intervals shown. For each recall metric, two-sided Mann-Whitney U test between HPC and CTX. Accuracy: consolidation time = 0 (p-value $= 1.004910 \cdot 10^{-4}$) and 12 h (p-value $= 1.333409 \cdot 10^{-4}$). True positive rate: consolidation time = 0 (p-value $= 1.004910 \cdot 10^{-4}$) and 12 h (p-value $= 1.277653 \cdot 10^{-4}$). False positive rate: consolidation time = 0 (p-value $= 1.407747 \cdot 10^{-4}$) and 12 h (p-value $= 2.194140 \cdot 10^{-4}$). Right: Population activity of engram cells encoding each stimulus in CTX (top) and HPC (bottom) (each color designates the engram cells encoding the respective stimulus in Fig. 1a; dashed line indicates threshold $\zeta^{thr} = 10$ Hz for engram cell activation). **b**, Top: CTX. Bottom: HPC. Left: Cumulative distribution of the total inhibitory synaptic weights onto individual excitatory engram cells at the end of training (black) and after 12 hours of consolidation (gray). Two-sided Kolmogorov-Smirnov test between the distribution of inhibitory weights onto excitatory engram cells at consolidation time = 0 and 12 h (CTX: p-value $= 1.202739 \cdot 10^{-8}$; HPC: p-value $= 6.573722 \cdot 10^{-125}$). Right: Population activity of excitatory neurons (dashed line indicates threshold $\zeta^{thr} = 10$ Hz for engram cell activation). **c-d**, Same as **a-b** but for an E/I ratio of 2 in both CTX and HPC. Recall curves: mean values and 90% confidence intervals shown. For each recall metric, two-sided Mann-Whitney U test between HPC and CTX. Accuracy: consolidation time = 0 (p-value $= 1.090950 \cdot 10^{-2}$) and 12 h (p-value $= 2.585758 \cdot 10^{-2}$). True positive rate: consolidation time = 0 (p-value $= 1.090950 \cdot 10^{-2}$) and 12 h (p-value $= 8.784000 \cdot 10^{-3}$). False positive rate: consolidation time = 0 (p-value $= 8.784000 \cdot 10^{-3}$) and 12 h (p-value $= 2.585758 \cdot 10^{-2}$). Cumulative distributions: two-sided Kolmogorov-Smirnov test between the distribution of inhibitory weights onto excitatory engram cells at consolidation time = 0 and 12 h (CTX: p-value = 0; HPC: p-value $= 7.165372 \cdot 10^{-127}$). **e-f**, Same as **a-b** but for an E/I ratio of 8 in both CTX and HPC. Recall curves: mean values and 90% confidence intervals shown. For each recall metric, two-sided Mann-Whitney U test between HPC and CTX. Accuracy: consolidation time = 0 (p-value $= 6.694381 \cdot 10^{-3}$) and 12 h (p-value $= 1.090950 \cdot 10^{-2}$). True positive rate: consolidation time = 0 (p-value $= 6.694381 \cdot 10^{-3}$) and 12 h (p-value $= 1.090950 \cdot 10^{-2}$). False positive rate: consolidation time = 0 (p-value $= 9.700785 \cdot 10^{-3}$) and 12 h (p-value $= 6.519642 \cdot 10^{-2}$). Cumulative distributions: two-sided Kolmogorov-Smirnov test between the distribution of inhibitory weights onto excitatory engram cells at consolidation time = 0 and 12 h (CTX: p-value = 0; HPC: p-value = 0). *p-value < 0.05 (see Methods). **a**, $n = 10$ trials. **c/e**, $n = 5$ trials. **a-f**, Network and simulation parameters as in Fig. 1b except that $N_{inh} = 2048$ and $T_{training} = 50$ min in **c-d** and that $N_{inh} = 512$ in **e-f**. Color as in Fig. 1a.

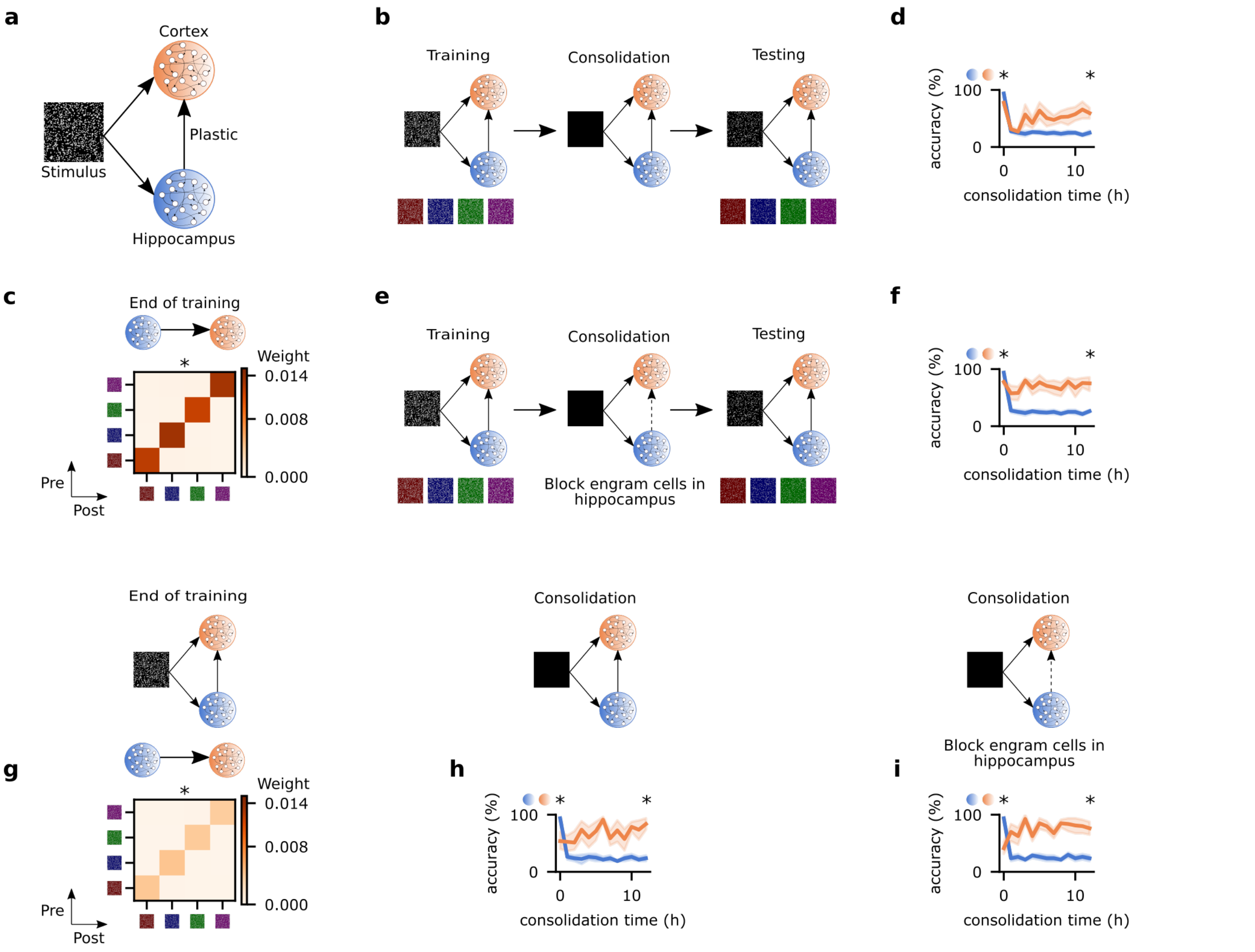

Supplementary Figure 6. Plastic monosynaptic HPC→CTX coupling has contradictory effect on CTX engram dynamics. **a**, Schematic of network model where HPC→CTX synapses are plastic. In Stimulus (STIM), light gray indicates active neurons firing above baseline whereas black indicates neurons at baseline firing rate. **b**, Schematic of simulation protocol with intact (control) HPC→CTX synapses for the network in **a**. **c**, Mean HPC→CTX weight strength at the end of training clustered according to engram cell preference (i.e., only mean weights between engram cells) for the network in **a** with $\lambda^{\beta}_{hpc\to ctx} = 85$ (see Methods). Two-sided Kolmogorov-Smirnov test between the distribution of HPC→CTX weights among engram cells encoding the same stimulus (i.e., diagonal) and that of HPC→CTX weights among engram cells encoding different stimuli (i.e., off-diagonal) at the end of training: p-value $= 0$. **d**, Memory recall accuracy in the testing phase of protocol **b** for the network in **c**. Two-sided Mann-Whitney U test between accuracy in HPC and CTX at consolidation time $= 0$ (p-value $= 5.980202 \cdot 10^{-5}$) and 12 h (p-value $= 5.100466 \cdot 10^{-4}$). **e**, Schematic of simulation protocol with the output of engram cells in HPC blocked during consolidation for the network in **a**. **f**, Memory recall accuracy in the testing phase of protocol **e** for the network in **c**. Two-sided Mann-Whitney U test between accuracy in HPC and CTX at consolidation time $= 0$ (p-value $= 5.980202 \cdot 10^{-5}$) and 12 h (p-value $= 1.285313 \cdot 10^{-3}$). **g**, Mean HPC→CTX weight strength at the end of training clustered according to engram cell preference (i.e., only mean weights between engram cells) for the network in **a** with $\lambda^{\beta}_{hpc\to ctx} = 170$ (see Methods). All other model parameters are the same as in **c**. Two-sided Kolmogorov-Smirnov test between the distribution of HPC→CTX weights among engram cells encoding the same stimulus (i.e., diagonal) and that of HPC→CTX weights among engram cells encoding different stimuli (i.e., off-diagonal) at the end of training: p-value $= 0$. **h**, Memory recall accuracy in the testing phase of protocol **b** for the network in **g**. Two-sided Mann-Whitney U test between accuracy in HPC and CTX at consolidation time $= 0$ (p-value $= 7.494958 \cdot 10^{-3}$) and 12 h (p-value $= 1.041777 \cdot 10^{-2}$). **i**, Memory recall accuracy in the testing phase of protocol **e** for the network in **g**. Two-sided Mann-Whitney U test between accuracy in HPC and CTX at consolidation time $= 0$ (p-value $= 7.494958 \cdot 10^{-3}$) and 12 h (p-value $= 1.066227 \cdot 10^{-2}$). *p-value < 0.05 (see Methods). **d/f**, $n = 10$ trials. **h/i**, $n = 5$ trials. **d/f/h-i**, Mean values and 90% confidence intervals shown. Color as in **a**. **b-c/e/g**, Stimuli as in Fig. 1a.

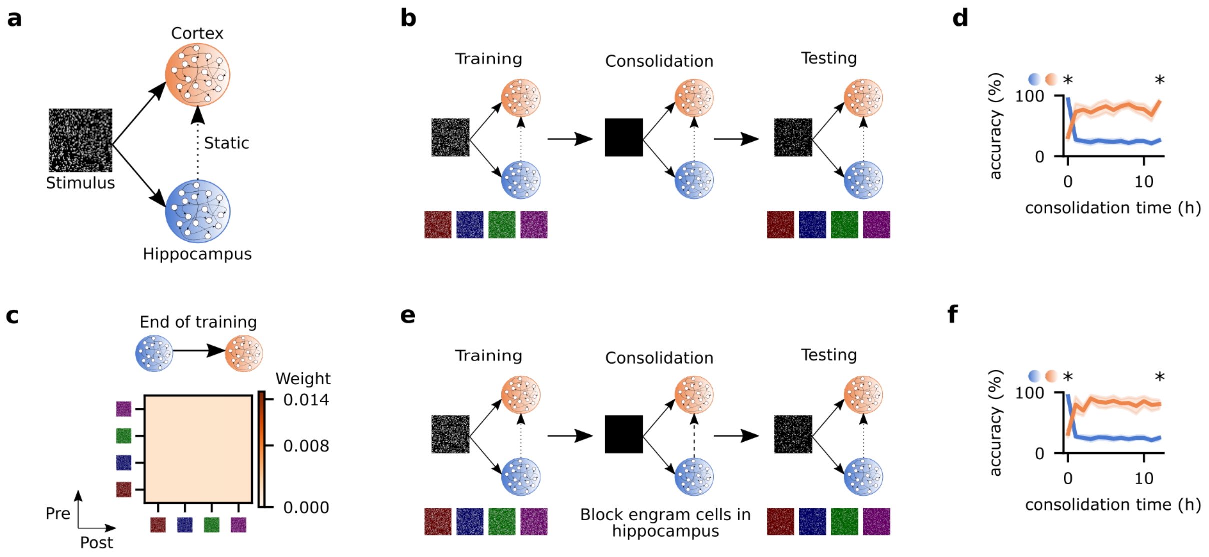

Supplementary Figure 7. Static HPC→CTX synapses render engram dynamics in CTX independent of HPC. **a**, Schematic of network model where HPC→CTX synapses are static. In Stimulus (STIM), light gray indicates active neurons firing above baseline whereas black indicates neurons at baseline firing rate. Network and simulation parameters are the same as in Supplementary Fig. 6a except that HPC→CTX synapses are static. **b**, Schematic of simulation protocol with intact (control) HPC→CTX synapses for the network in **a**. **c**, Mean HPC→CTX weight strength at the end of training clustered according to engram cell preference (i.e., only mean weights between engram cells) for the network in **a**. Two-sided Kolmogorov-Smirnov test between the distribution of HPC→CTX weights among engram cells encoding the same stimulus (i.e., diagonal) and that of HPC→CTX weights among engram cells encoding different stimuli (i.e., off-diagonal) at the end of training: p-value = 1. **d**, Memory recall accuracy in the testing phase of protocol **b**. Two-sided Mann-Whitney U test between accuracy in HPC and CTX at consolidation time = 0 (p-value $= 5.892694 \cdot 10^{-5}$) and 12 h (p-value $= 1.285509 \cdot 10^{-4}$). **e**, Schematic of simulation protocol with the output of engram cells in HPC blocked during consolidation for the network in **a**. **f**, Memory recall accuracy in the testing phase of protocol **e**. Two-sided Mann-Whitney U test between accuracy in HPC and CTX at consolidation time = 0 (p-value $= 5.892694 \cdot 10^{-5}$) and 12 h (p-value $= 1.349668 \cdot 10^{-4}$). *p-value < 0.05 (see Methods). **d/f**, $n = 10$ trials. Mean values and 90% confidence intervals shown. Color as in **a**. **b-c/e**, Stimuli as in Fig. 1a.

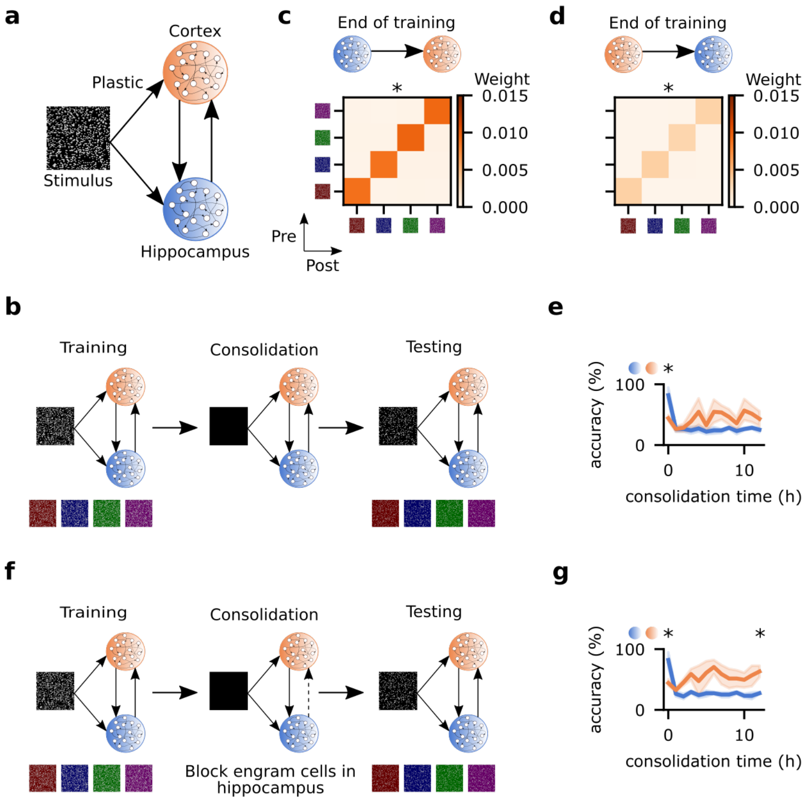

Supplementary Figure 8. Bidirectional HPC↔CTX synapses lead to inconsistent engram dynamics in CTX. **a**, Schematic of network model with plastic HPC→CTX and CTX→HPC synapses. In Stimulus (STIM), light gray indicates active neurons firing above baseline whereas black indicates neurons at baseline firing rate. Network and simulation parameters are the same as in Supplementary Fig. 6a except that $\lambda^{\beta}_{hpc\to ctx} = 100$ and $T_{training} = 35$ min. Parameters for CTX→HPC are the same as HPC→CTX except $\epsilon_{ctx\to hpc} = 0.01$ (see Methods). **b**, Schematic of simulation protocol with intact (control) HPC→CTX synapses for the network in **a**. **c**, Mean HPC→CTX weight strength at the end of training clustered according to engram cell preference (i.e., only mean weights between engram cells) for the network in **a**. Two-sided Kolmogorov-Smirnov test between the distribution of HPC→CTX weights among engram cells encoding the same stimulus (i.e., diagonal) and that of HPC→CTX weights among engram cells encoding different stimuli (i.e., off-diagonal) at the end of training: p-value = 0. **d**, Mean CTX→HPC weight strength at the end of training clustered according to engram cell preference (i.e., only mean weights between engram cells) for the network in **a**. Two-sided Kolmogorov-Smirnov test between the distribution of CTX→HPC weights among engram cells encoding the same stimulus (i.e., diagonal) and that of CTX→HPC weights among engram cells encoding different stimuli (i.e., off-diagonal) at the end of training: p-value $= 8.814715 \cdot 10^{-261}$. **e**, Memory recall accuracy in the testing phase of protocol **b**. Two-sided Mann-Whitney U test between accuracy in HPC and CTX at consolidation time = 0 (p-value $= 1.849078 \cdot 10^{-2}$) and 12 h (p-value $= 5.165175 \cdot 10^{-2}$). **f**, Schematic of simulation protocol with the output of engram cells in HPC blocked during consolidation for the network in **a**. **g**, Memory recall accuracy in the testing phase of protocol **f**. Two-sided Mann-Whitney U test between accuracy in HPC and CTX at consolidation time = 0 (p-value $= 1.849078 \cdot 10^{-2}$) and 12 h (p-value $= 9.700785 \cdot 10^{-3}$). *p-value < 0.05 (see Methods). **e/g**, $n = 5$ trials. Mean values and 90% confidence intervals shown. Color as in **a**. **b/c-d/f**, Stimuli as in Fig. 1a.

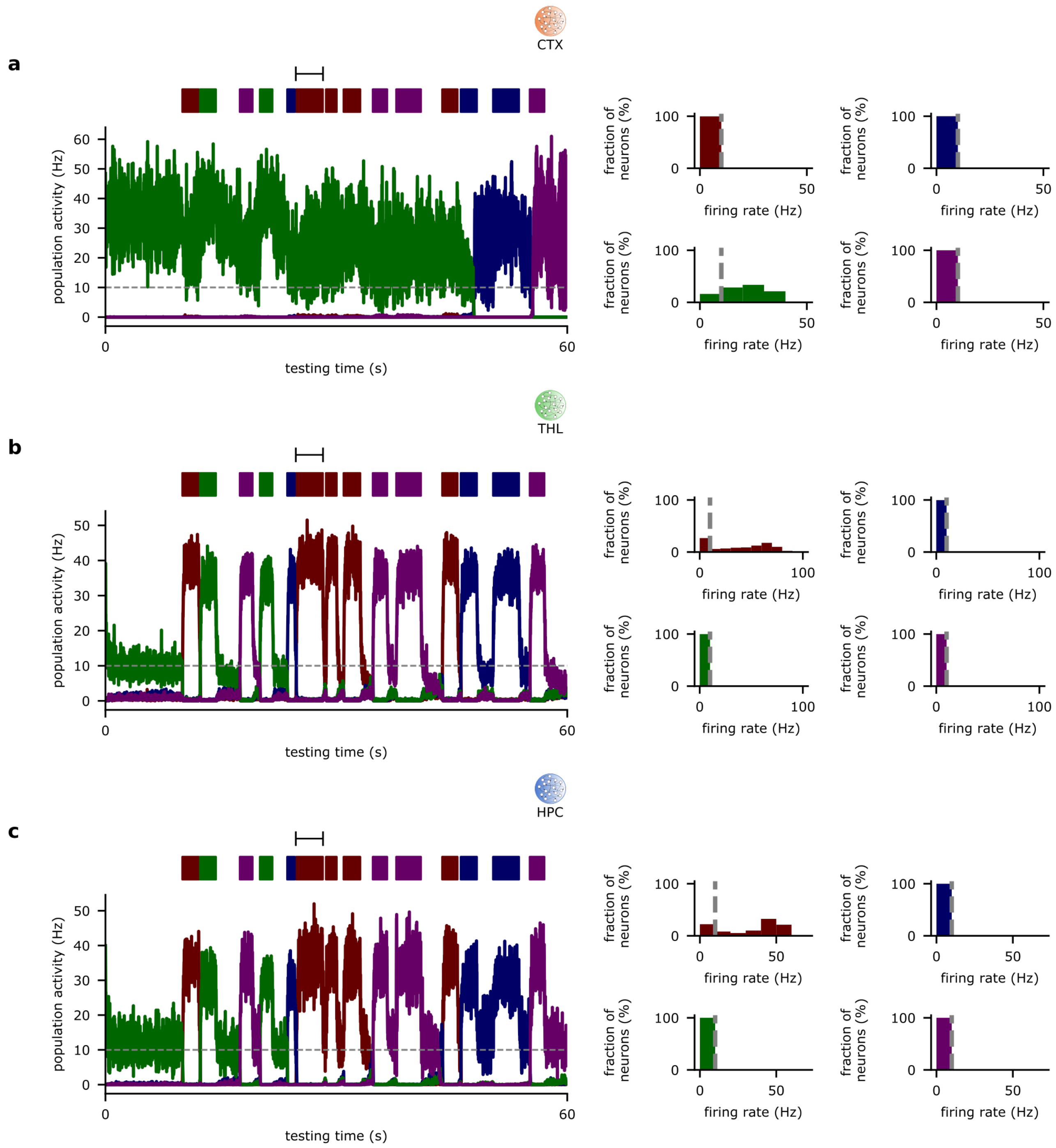

Supplementary Figure 9. Recent memory recall relies on HPC engram cells. Analysis of recent memory recall in Fig. 2b. **a**, Left: Population activity of CTX excitatory engram cells in the testing phase immediately following training (i.e., prior to consolidation) with cue presentation times displayed at the top. Right: histograms of the firing rates of CTX excitatory engram cells encoding each stimulus for the cue presentation interval marked in the activity plot on the left. Each color in activity plots and histograms designates the engram cells encoding the respective stimulus in Fig. 2a. Dashed line in activity plots and histograms indicates threshold $\zeta^{thr} = 10$ Hz for engram cell activation. **b**, Same as **a** but for THL. **c**, Same as **a** but for HPC. **a-c**, Color as in Fig. 2a.

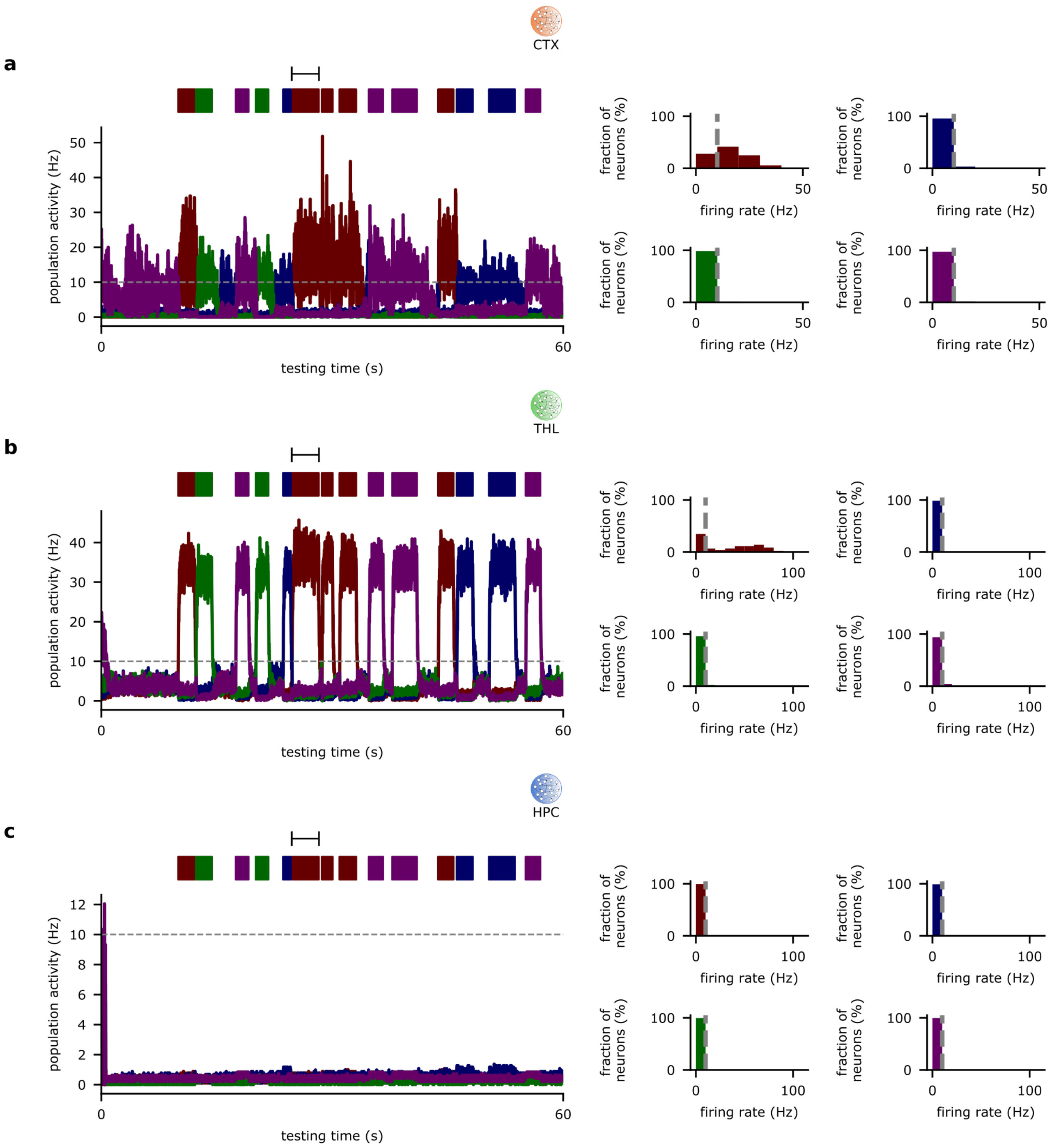

Supplementary Figure 10. Remote memory recall relies on CTX engram cells. Analysis of remote memory recall in Fig. 2b. **a**, Left: Population activity of CTX excitatory engram cells in the testing phase after 24 hours of consolidation with cue presentation times displayed at the top. Right: histograms of the firing rates of CTX excitatory engram cells encoding each stimulus for the cue presentation interval marked in the activity plot on the left. Each color in activity plots and histograms designates the engram cells encoding the respective stimulus in Fig. 2a. Dashed line in activity plots and histograms indicates threshold $\zeta^{thr} = 10$ Hz for engram cell activation. **b**, Same as **a** but for THL. **c**, Same as **a** but for HPC. **a-c**, Color as in Fig. 2a.

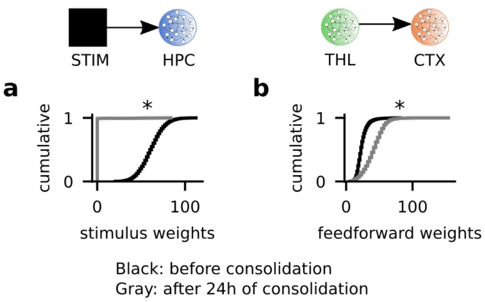

Supplementary Figure 11. Feedforward weight changes underlie engram cell state transitions in HPC and CTX. Analysis of STIM→HPC and THL→CTX feedforward weights in Fig. 2b. **a**, Cumulative distribution of the total STIM→HPC feedforward synaptic weights onto individual excitatory engram cells at the end of training and after 24 hours of consolidation. Two-sided Kolmogorov-Smirnov test between the distribution of STIM→HPC weights onto excitatory engram cells at the end of training and after 24 h of consolidation: p-value = 0. **b**, Same as **a** but for THL→CTX feedforward synaptic weights. Two-sided Kolmogorov-Smirnov test between the distribution of THL→CTX weights onto excitatory engram cells at the end of training and after 24 h of consolidation: p-value = 0. *p-value $<$ 0.05 (see Methods).

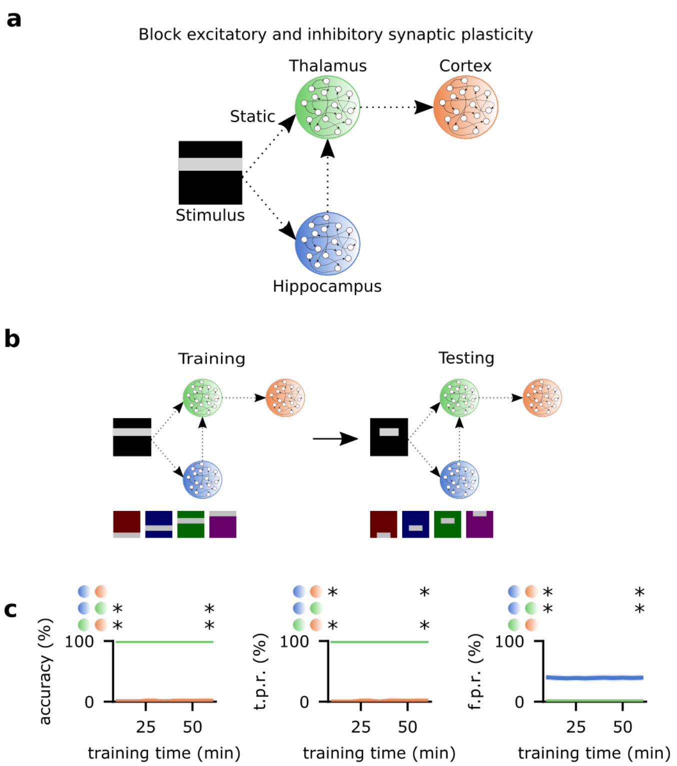

Supplementary Figure 12. Excitatory and inhibitory synaptic plasticity are essential for memory acquisition. **a**, Schematic of network model with excitatory and inhibitory synaptic plasticity blocked in the entire network. In Stimulus (STIM), light gray indicates active neurons firing above baseline whereas black indicates neurons at baseline firing rate. Network and simulation parameters are the same as in Fig. 2a except that all synapses are static. **b**, Schematic of simulation protocol. Training and testing stimuli as in Fig. 2a. **c**, Memory recall in the testing phase as a function of training time. Recall curves (from left to right): accuracy, true positive rate, and false positive rate. Color as in **a**. $n = 5$ trials. Mean values and 90% confidence intervals shown. For each recall metric, two-sided Mann-Whitney U test. Accuracy: training time = 10 (HPC vs. CTX p-value $= 0.920340$; HPC vs. THL p-value $= 3.976752 \cdot 10^{-3}$; THL vs. CTX p-value $= 3.976752 \cdot 10^{-3}$) and 60 min (HPC vs. CTX p-value $= 0.423711$; HPC vs. THL p-value $= 3.976752 \cdot 10^{-3}$; THL vs. CTX p-value $= 5.583617 \cdot 10^{-3}$). True positive rate: training time = 10 (HPC vs. CTX p-value $= 3.976752 \cdot 10^{-3}$; HPC vs. THL p-value $= 0.920340$; THL vs. CTX p-value $= 3.976752 \cdot 10^{-3}$) and 60 min (HPC vs. CTX p-value $= 5.583617 \cdot 10^{-3}$; HPC vs. THL p-value $= 0.920340$; THL vs. CTX p-value $= 5.583617 \cdot 10^{-3}$). False positive rate: training time = 10 (HPC vs. CTX p-value $= 7.290358 \cdot 10^{-3}$; HPC vs. THL p-value $= 7.290358 \cdot 10^{-3}$; THL vs. CTX p-value $= 0.920340$) and 60 min (HPC vs. CTX p-value $= 7.290358 \cdot 10^{-3}$; HPC vs. THL p-value $= 7.290358 \cdot 10^{-3}$; THL vs. CTX p-value $= 0.920340$). *p-value < 0.05 (see Methods).

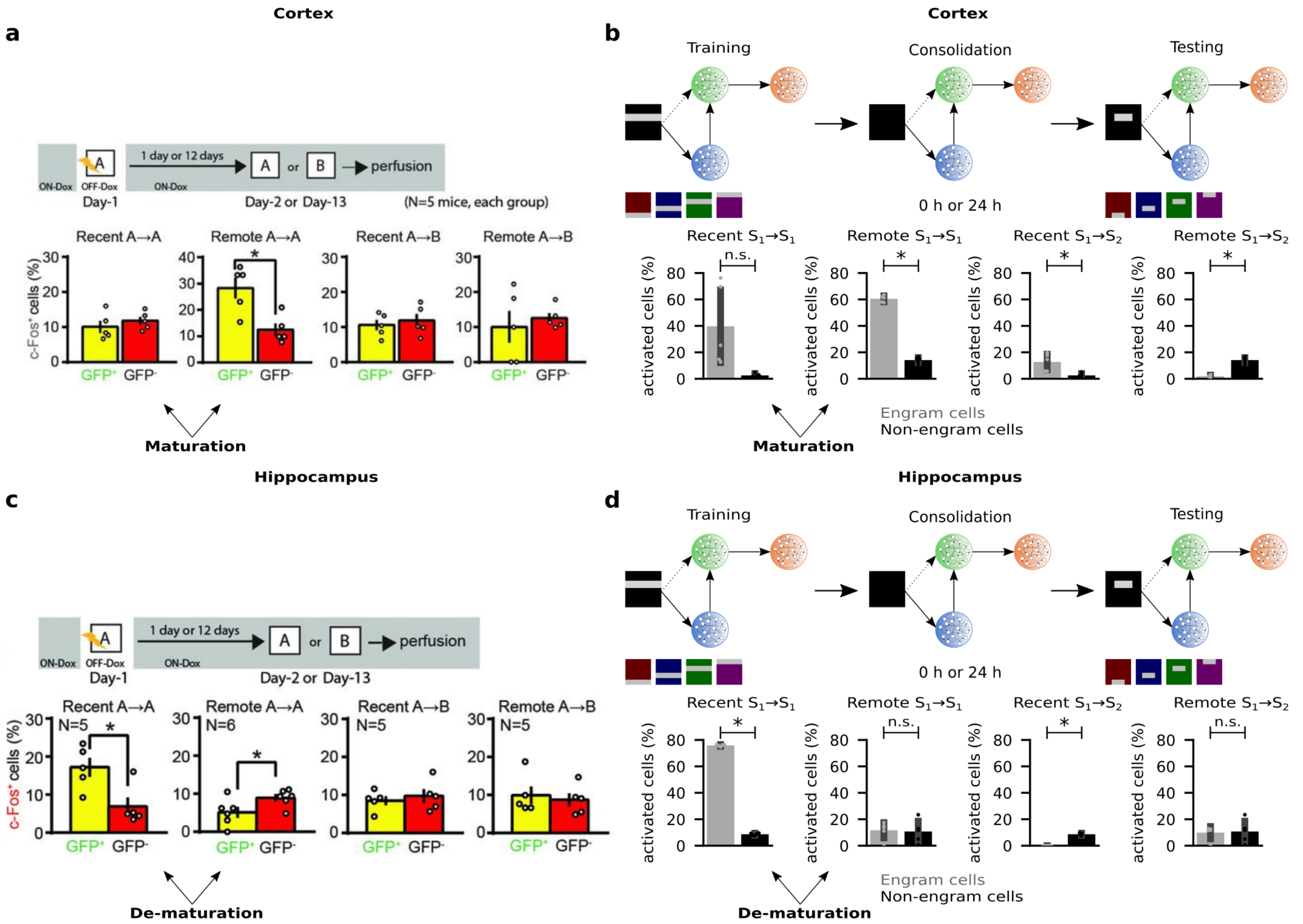

Supplementary Figure 13. Engram cell dynamics in the model are consistent with previous experiments. Engram cell maturation in cortex (**a** experiments, **b** model) and de-maturation in hippocampus (**c** experiments, **d** model). **a**, Top: Experimental schedule from Kitamura et al. 2017. Mice are fear conditioned in Context A on Day-1 (training) and then are placed back in Context A or in a novel Context B either on Day-2 or Day-13 (testing). Bottom: percentage of c-Fos$^+$ cells in H2B-GFP$^+$ and H2B-GFP$^-$ cells in medial prefrontal cortex. c-Fos$^+$ cells are activated during recall. H2B-GFP$^+$ are engram cells activated during training while H2B-GFP$^-$ cells are non-engram cells that were not activated during training. Recent denotes a delay of 1 day between training and testing. Remote denotes a delay of 12 days between training and testing. Error bars show standard error of the mean (SEM). Unpaired t-test between the percentages of c-Fos$^+$ cells in H2B-GFP$^+$ and H2B-GFP$^-$ cells. **b**, Top: Simulation protocol (same as Fig. 2b). Bottom: percentage of engram (gray) and non-engram (black) cells in CTX activated during testing in Fig. 2c. Recent denotes a delay of 0 h (i.e., no consolidation time) between training and testing. Remote denotes a delay of 24 h of consolidation between training and testing. $S_1 \to S_1$ indicates the percentage of activated cells when partial cues of the stimulus encoded by an engram cell ensemble are presented in the testing phase, averaged over all engram cell ensembles. $S_1 \to S_2$ indicates the percentage of activated cells when partial cues of stimuli other than the one encoded by an engram cell ensemble are presented in the testing phase, averaged over all engram cell ensembles. $n = 5$ trials. Error bars show standard deviation. Unpaired t-test (UT), Welch's t-test (WT), or Mann-Whitney U test (MT) between the percentages of engram cells and non-engram cells activated during testing (see Methods). Recent $S_1 \to S_1$: WT p-value $= 5.387418 \cdot 10^{-2}$. Remote $S_1 \to S_1$: UT p-value $= 1.057208 \cdot 10^{-9}$. Recent $S_1 \to S_2$: WT p-value $= 2.445840 \cdot 10^{-2}$. Remote $S_1 \to S_2$: UT p-value $= 1.992456 \cdot 10^{-6}$. **c**, Same as **a** but for cells in the hippocampal dentate gyrus. Unpaired t-test between the percentages of c-Fos$^+$ cells in H2B-GFP$^+$ and H2B-GFP$^-$ cells. **d**, Same as **b** but for cells in HPC. Unpaired t-test (UT), Welch's t-test (WT), or Mann-Whitney U test (MT) between the percentages of engram cells and non-engram cells activated during testing (see Methods). Recent $S_1 \to S_1$: UT p-value $= 1.207993 \cdot 10^{-14}$. Remote $S_1 \to S_1$: UT p-value $= 0.861536$. Recent $S_1 \to S_2$: MT p-value $= 9.700785 \cdot 10^{-3}$. Remote $S_1 \to S_2$: MT p-value $= 1$. *p-value < 0.05 (see Methods). n.s., not significant. **a/c**, From Takashi Kitamura, Sachie K Ogawa, Dheeraj S Roy, Teruhiro Okuyama, Mark D Morrissey, Lillian M Smith, Roger L Redondo, and Susumu Tonegawa. Engrams and circuits crucial for systems consolidation of a memory. *Science*, 356(6333):73–78, 2017. `https://doi.org/10.1126/science.aam6808`. Reprinted with permission from AAAS.

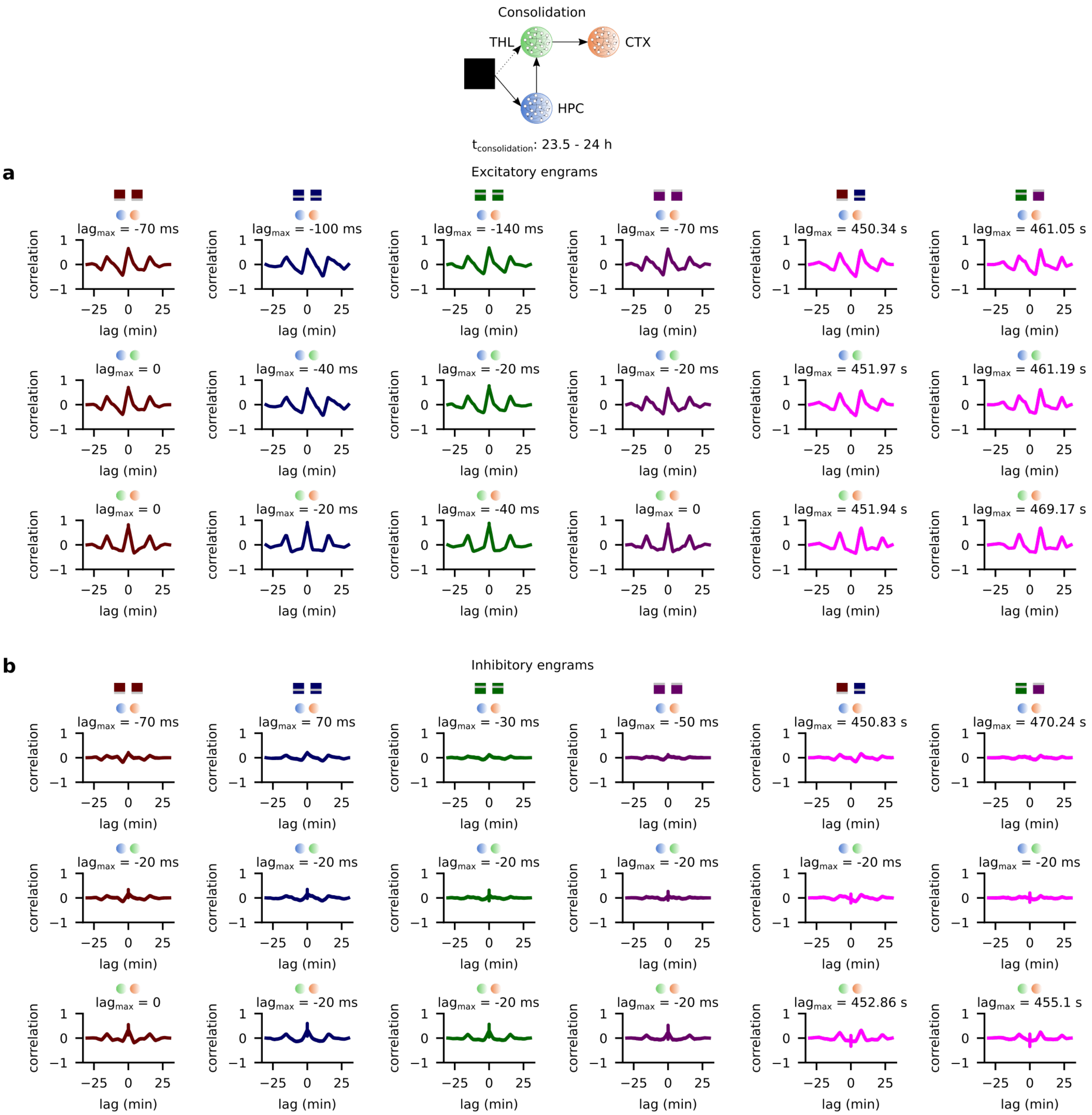

Supplementary Figure 14. Coupled reactivations of excitatory and inhibitory engrams in the HPC→THL→CTX circuit. a, Cross-correlograms of the population activities of excitatory engram cells in two different regions during the consolidation phase of Fig. 2c (see Methods for the definition of lag_{max}). Pair of stimuli indicated at the top. Pair of regions indicated at the top of each cross-correlogram. Time interval: between 23.5 and 24 h of consolidation. **b**, Same as **a** but for the population activities of inhibitory engram cells. **a-b**, Color and stimuli as in Fig. 2a.

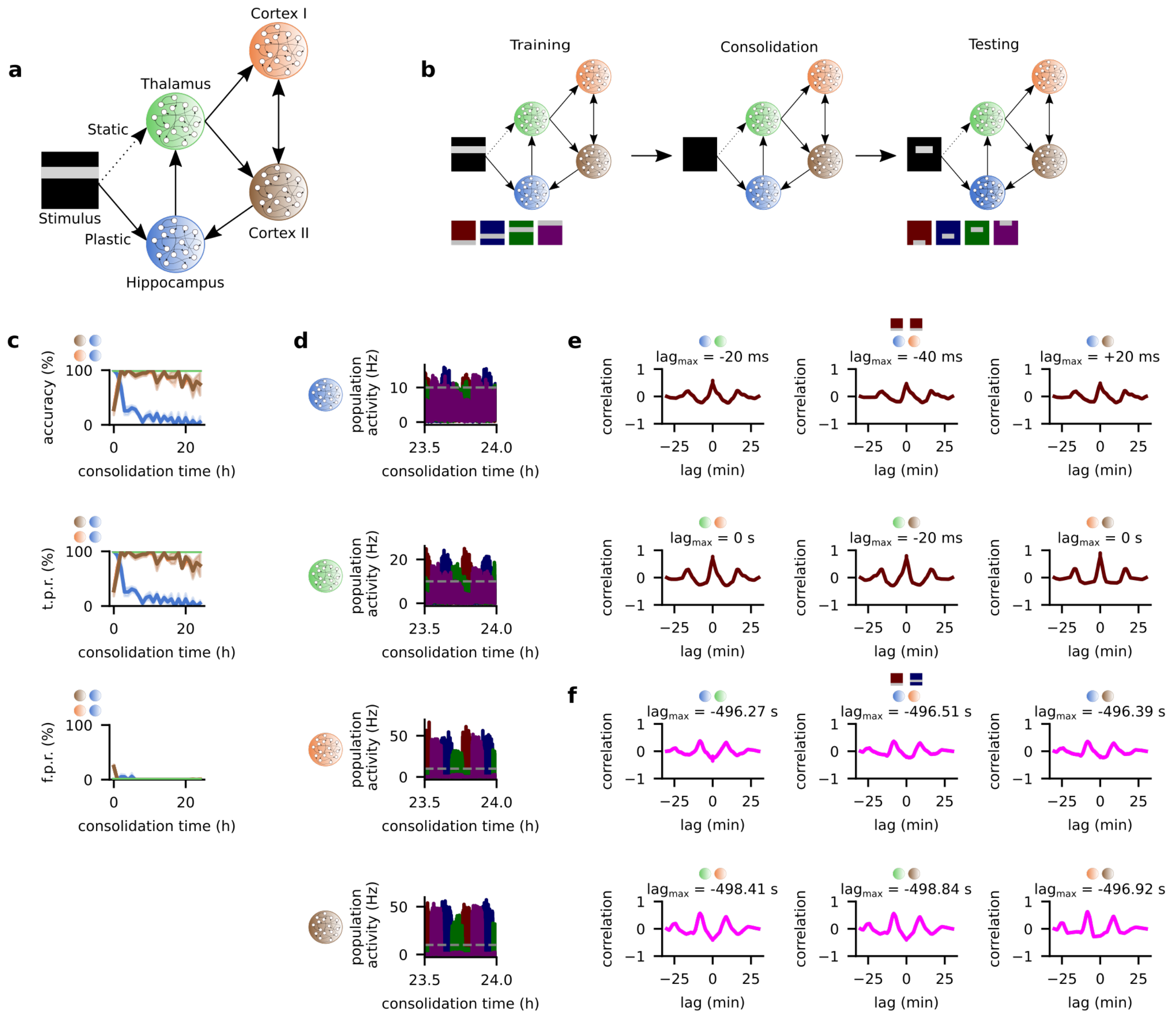

Supplementary Figure 15. Circuit with multiple cortical regions, reentrant connectivity, and bidirectional coupling exhibits consistent engram dynamics. **a**, Schematic of network model with STIM, HPC, THL, CTX I, and CTX II. Colors as in Fig. 2a. STIM→THL synapses are static but the remaining feedforward projections are plastic as in Fig. 2a. **b**, Schematic of simulation protocol. Stimuli as in Fig. 2a. **c**, Memory recall in the testing phase as a function of consolidation time. Top to bottom: accuracy, true positive rate, and false positive rate. $n = 3$ trials. Mean values and 90% confidence intervals shown. For each recall metric, two-sided Mann-Whitney U test. Accuracy: consolidation time = 0 (CTX I vs. HPC p-value = 0.063603; CTX II vs. HPC p-value = 0.063603) and 24 h (CTX I vs. HPC p-value = 0.076523; CTX II vs. HPC p-value = 0.076523). True positive rate: consolidation time = 0 (CTX I vs. HPC p-value = 0.063603; CTX II vs. HPC p-value = 0.063603) and 24 h (CTX I vs. HPC p-value = 0.076523; CTX II vs. HPC p-value = 0.076523). False positive rate: consolidation time = 0 (CTX I vs. HPC p-value = 0.063603; CTX II vs. HPC p-value = 0.063603) and 24 h (CTX I vs. HPC p-value = 0.504985; CTX II vs. HPC p-value = 0.504985). **d**, Population activity of excitatory engram cells in the consolidation phase. Top to bottom: HPC, THL, CTX I, and CTX II (dashed line indicates threshold $\zeta^{thr} = 10$ Hz for engram cell activation). **e**, Cross-correlograms of the population activities of excitatory engram cells encoding the same stimulus in two different regions in the consolidation interval shown in **d**. Pair of stimuli indicated at the top. Pair of regions indicated at the top of each cross-correlogram (see Methods for the definition of $\mathrm{lag}_{\mathrm{max}}$). **f**, Same as **e** but for excitatory engram cells encoding different stimuli in two different regions. *p-value < 0.05 (see Methods). **b-f**, Color as in **a**. Network and simulation parameters are the same as in Fig. 2a except that $\epsilon_{thl \to ctxI} = \epsilon_{thl \to ctxII} = 0.05$, $w_{thl \to ctxI} = w_{thl \to ctxII} = 0.2$, $\eta^{exc}_{thl \to ctxI} = \eta^{exc}_{thl \to ctxII} = 10^{-3}$, $\lambda^{\beta}_{thl \to ctxI} = \lambda^{\beta}_{thl \to ctxII} = 85$, $\tau^{cons}_{thl \to ctxI} = \tau^{cons}_{thl \to ctxII} = 20$ min, $\epsilon_{ctxI \to ctxII} = \epsilon_{ctxII \to ctxI} = 0.01$, $w_{ctxI \to ctxII} = w_{ctxII \to ctxI} = 0.05$, $\eta^{exc}_{ctxI \to ctxII} = \eta^{exc}_{ctxII \to ctxI} = 10^{-3}$, $\lambda^{\beta}_{ctxI \to ctxII} = \lambda^{\beta}_{ctxII \to ctxI} = 50$, $\tau^{cons}_{ctxI \to ctxII} = \tau^{cons}_{ctxII \to ctxI} = 20$ min, $\epsilon_{ctxII \to hpc} = 0.01$, $w_{ctxII \to hpc} = 0.05$, $\eta^{exc}_{ctxII \to hpc} = 10^{-3}$, $\lambda^{\beta}_{ctxII \to hpc} = 50$, $\tau^{cons}_{ctxII \to hpc} = 20$ min, $w^{max}_{ctxII \to hpc} = 0.5$, $\nu^{con}_{ext \to ctxI} = \nu^{con}_{ext \to ctxII} = 0$, $\nu^{con}_{ext \to hpc} = 0.75$ Hz, and $T_{training} = 25$ min.

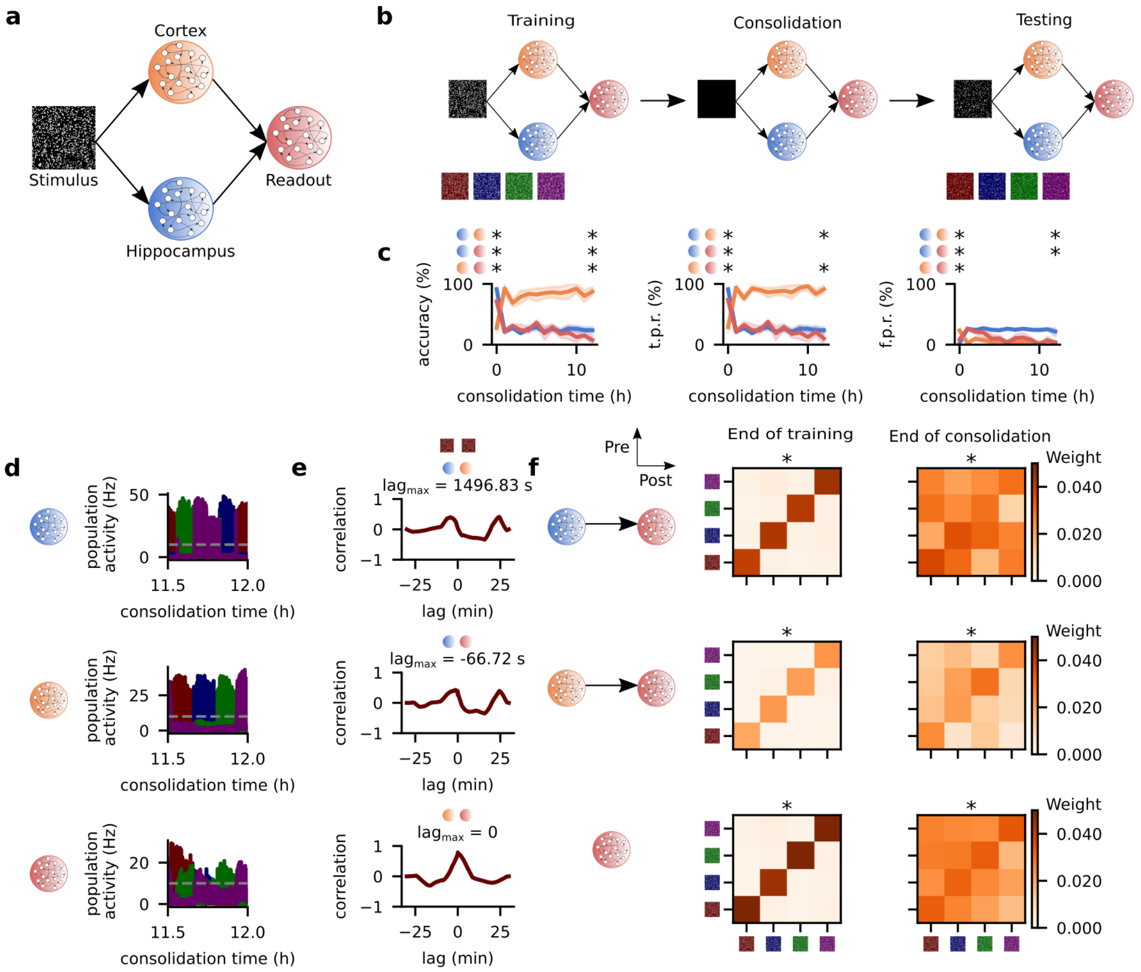

Supplementary Figure 16. Decoupled engram reactivations fail to sustain recall in a readout region. **a**, Schematic of network model with Stimulus (STIM), Hippocampus (HPC), Cortex (CTX), and Readout (RDT). In STIM, light gray indicates active neurons firing above baseline whereas black indicates neurons at baseline firing rate. **b**, Schematic of simulation protocol. Stimuli as in Fig. 1a. Network simulation as in Fig. 1b. **c**, Memory recall in the testing phase as a function of consolidation time. Left to right: accuracy, true positive rate, and false positive rate. $n = 5$ trials. Mean values and 90% confidence intervals shown. For each recall metric, two-sided Mann-Whitney U test. Accuracy: consolidation time = 0 (HPC vs. CTX p-value $= 1.066227 \cdot 10^{-2}$; HPC vs. RDT p-value $= 1.066227 \cdot 10^{-2}$; CTX vs. RDT p-value $= 1.166731 \cdot 10^{-2}$) and 12 h (HPC vs. CTX p-value $= 9.937010 \cdot 10^{-3}$; HPC vs. RDT p-value $= 4.125002 \cdot 10^{-2}$; CTX vs. RDT p-value $= 9.937010 \cdot 10^{-3}$). True positive rate: consolidation time = 0 (HPC vs. CTX p-value $= 1.066227 \cdot 10^{-2}$; HPC vs. RDT p-value $= 1.041777 \cdot 10^{-2}$; CTX vs. RDT p-value $= 1.141204 \cdot 10^{-2}$) and 12 h (HPC vs. CTX p-value $= 1.041777 \cdot 10^{-2}$; HPC vs. RDT p-value $= 0.150282$; CTX vs. RDT p-value $= 1.066227 \cdot 10^{-2}$). False positive rate: consolidation time = 0 (HPC vs. CTX p-value $= 1.090950 \cdot 10^{-2}$; HPC vs. RDT p-value $= 1.017601 \cdot 10^{-2}$; CTX vs. RDT p-value $= 1.090950 \cdot 10^{-2}$) and 12 h (HPC vs. CTX p-value $= 4.321959 \cdot 10^{-2}$; HPC vs. RDT p-value $= 2.408555 \cdot 10^{-2}$; CTX vs. RDT p-value $= 0.515181$). **d**, Population activity of excitatory engram cells in the consolidation phase. Top to bottom: HPC, CTX, and RDT (each color designates the engram cells encoding the respective stimulus in Fig. 1a; dashed line indicates threshold $\zeta^{thr} = 10$ Hz for engram cell activation). **e**, Cross-correlograms of the population activities of excitatory engram cells encoding the same stimulus in two different regions in the consolidation interval shown in **d**. Pair of stimuli indicated at the top. Pair of regions indicated at the top of each cross-correlogram (see Methods for the definition of lag_{max}). **f**, Mean weight strength of excitatory synapses onto excitatory neurons clustered according to engram cell preference for the network in **a**. Top to bottom: HPC→RDT (feedforward), CTX→RDT (feedforward), and RDT (recurrent). Left: end of the training phase. Right: after 12 hours of consolidation. Two-sided Kolmogorov-Smirnov test between the distribution of excitatory weights among engram cells encoding the same stimulus (i.e., diagonal) and that of excitatory weights among engram cells encoding different stimuli (i.e., off-diagonal) at the end of training (HPC→RDT: p-value = 0; CTX→RDT: p-value = 0; RDT: p-value = 0) and after 12 h of consolidation (HPC→RDT: p-value = 0; CTX→RDT: p-value = 0; RDT: p-value = 0). *p-value < 0.05 (see Methods). **b-f**, Color as in **a**.

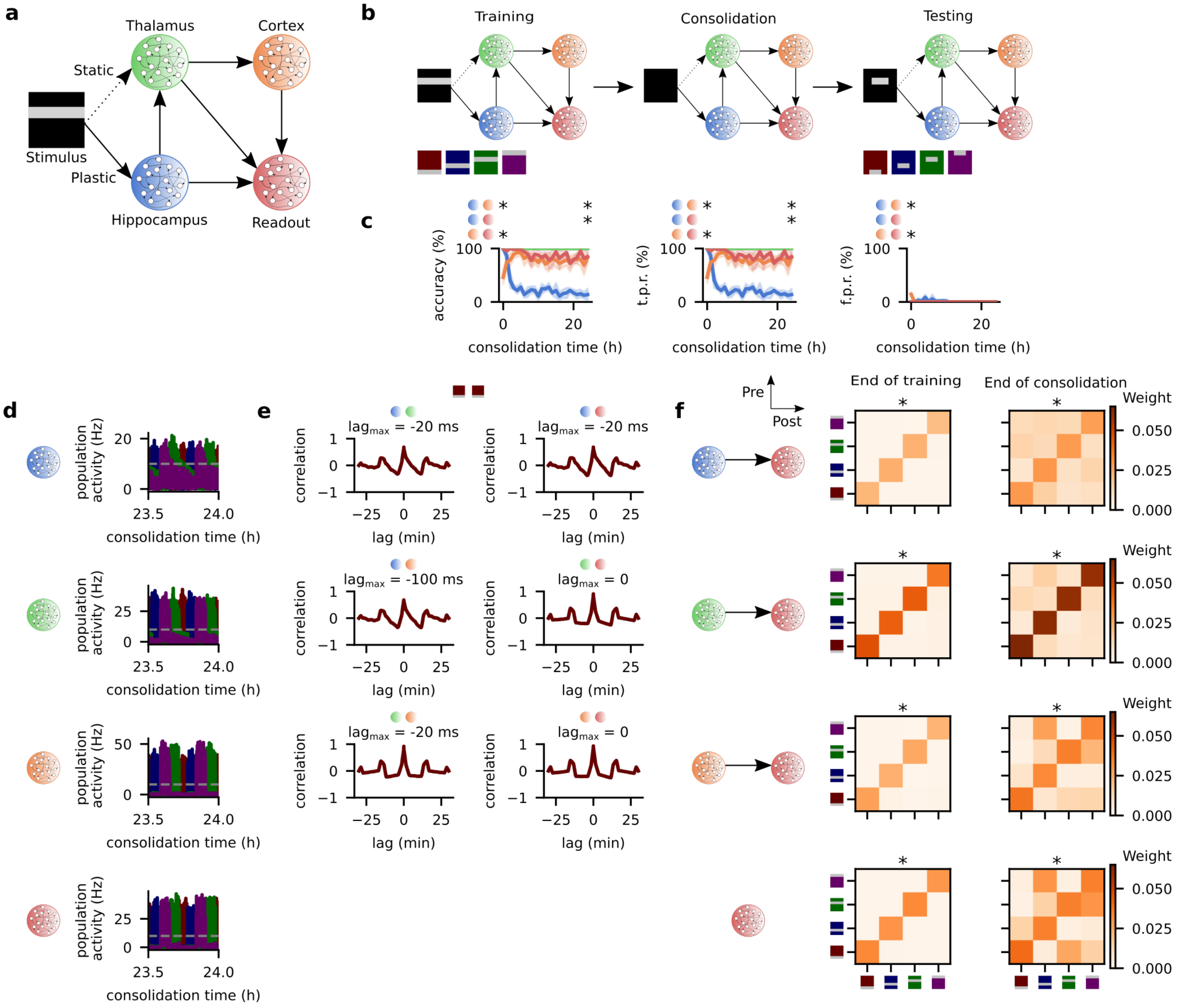

Supplementary Figure 17. Coupled engram reactivations enable stable recall in a readout region. **a**, Schematic of network model with Stimulus (STIM), Hippocampus (HPC), Thalamus (THL), Cortex (CTX), and Readout (RDT). In STIM, light gray indicates active neurons firing above baseline whereas black indicates neurons at baseline firing rate. **b**, Schematic of simulation protocol. Stimuli as in Fig. 2a. Network simulation as in Fig. 2b. **c**, Memory recall in the testing phase as a function of consolidation time. Left to right: accuracy, true positive rate, and false positive rate. $n = 5$ trials. Mean values and 90% confidence intervals shown. For each recall metric, two-sided Mann-Whitney U test. Accuracy / true positive rate (same value for both metrics in each case): consolidation time = 0 (HPC vs. CTX p-value = $7.290358 \cdot 10^{-3}$; HPC vs. RDT p-value = 0.920340; CTX vs. RDT p-value = $7.290358 \cdot 10^{-3}$) and 24 h (HPC vs. CTX p-value = $1.166731 \cdot 10^{-2}$; HPC vs. RDT p-value = $1.115943 \cdot 10^{-2}$; CTX vs. RDT p-value = 1). False positive rate: consolidation time = 0 (HPC vs. CTX p-value = $7.494958 \cdot 10^{-3}$; HPC vs. RDT p-value = 0.920340; CTX vs. RDT p-value = $7.494958 \cdot 10^{-3}$) and 24 h (HPC vs. CTX p-value = 0.920340; HPC vs. RDT p-value = 0.920340; CTX vs. RDT p-value = 0.920340). **d**, Population activity of excitatory engram cells in the consolidation phase. Top to bottom: HPC, THL, CTX, and RDT (each color designates the engram cells encoding the respective stimulus in Fig. 2a; dashed line indicates threshold $\zeta^{thr} = 10$ Hz for engram cell activation). **e**, Cross-correlograms of the population activities of excitatory engram cells encoding the same stimulus in two different regions in the consolidation interval shown in **d**. Pair of stimuli indicated at the top. Pair of regions indicated at the top of each cross-correlogram (see Methods for the definition of lag_{max}). **f**, Mean weight strength of excitatory synapses onto excitatory neurons clustered according to engram cell preference for the network in **a**. Top to bottom: HPC→RDT (feedforward), THL→RDT (feedforward), CTX→RDT (feedforward), and RDT (recurrent). Left: end of the training phase. Right: after 24 hours of consolidation. Two-sided Kolmogorov-Smirnov test between the distribution of excitatory weights among engram cells encoding the same stimulus (i.e., diagonal) and that of excitatory weights among engram cells encoding different stimuli (i.e., off-diagonal) at the end of training (HPC→RDT: p-value = 0; THL→RDT: p-value = 0; CTX→RDT: p-value = 0; RDT: p-value = 0) and after 24 h of consolidation (HPC→RDT: p-value = 0; THL→RDT: p-value = 0; CTX→RDT: p-value = 0; RDT: p-value = 0). *p-value < 0.05 (see Methods). **b-f**, Color as in **a**.

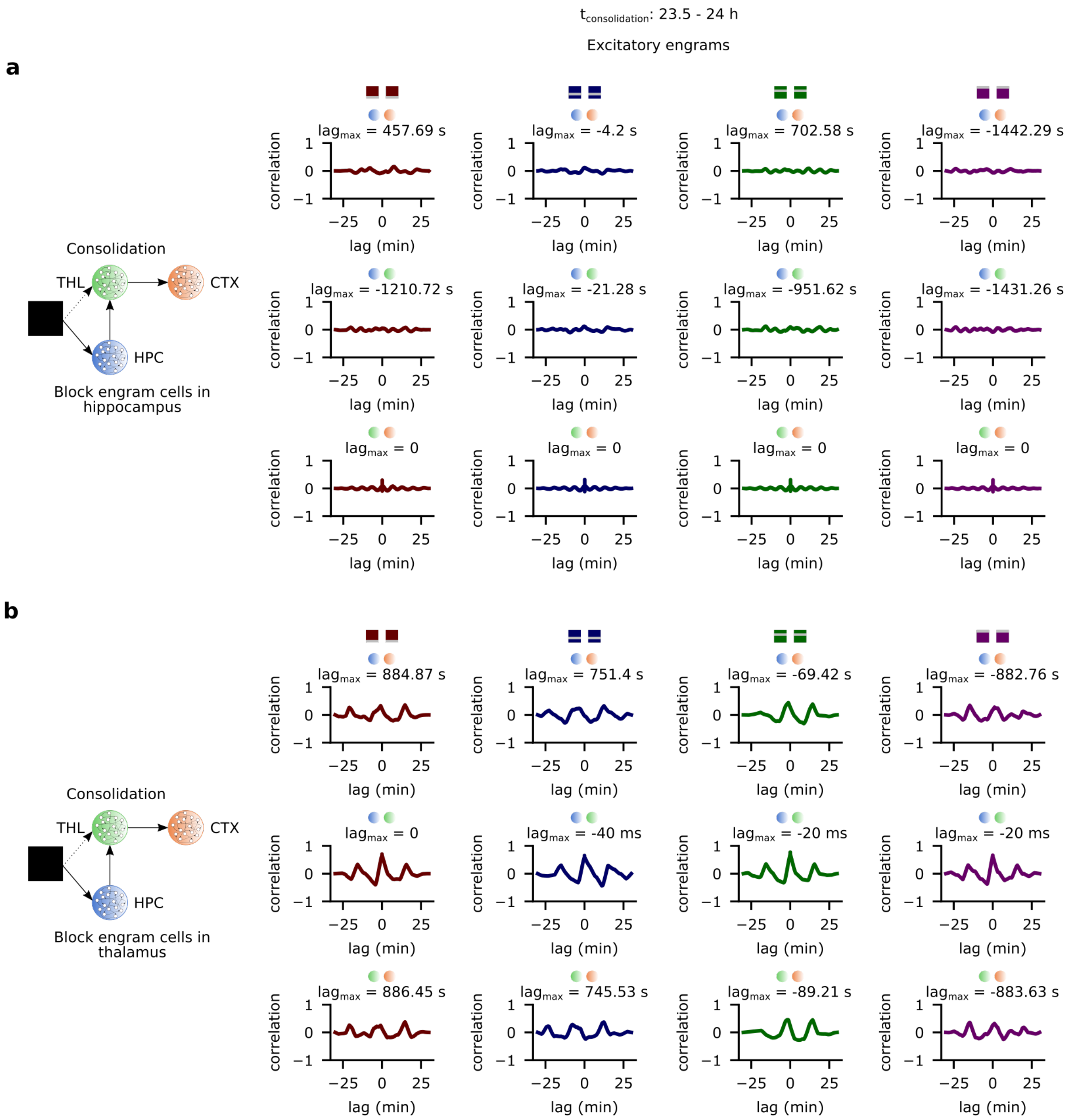

Supplementary Figure 18. Blocking excitatory engram cells in either HPC or THL leads to decoupled engram reactivations in the HPC→THL→CTX circuit. a, Cross-correlograms of the population activities of excitatory engram cells in two different regions during the consolidation phase of Fig. 2d (see Methods for the definition of lag_{max}). Pair of stimuli indicated at the top. Pair of regions indicated at the top of each cross-correlogram. Time interval: between 23.5 and 24 h of consolidation. **b**, Same as **a** but for Fig. 2e. Note that blocking engram cells in HPC during consolidation prevents alternating engram reactivations in THL and CTX (Fig. 2d) but blocking engram cells in THL during consolidation does not (Fig. 2e). This leads to flatter cross-correlograms when blocking HPC engram cells (**a**) compared to blocking THL engram cells (**b**) since only in the latter case it is possible to find a lag for which the correlation between engram activity pairs is high. **a-b**, Color and stimuli as in Fig. 2a.

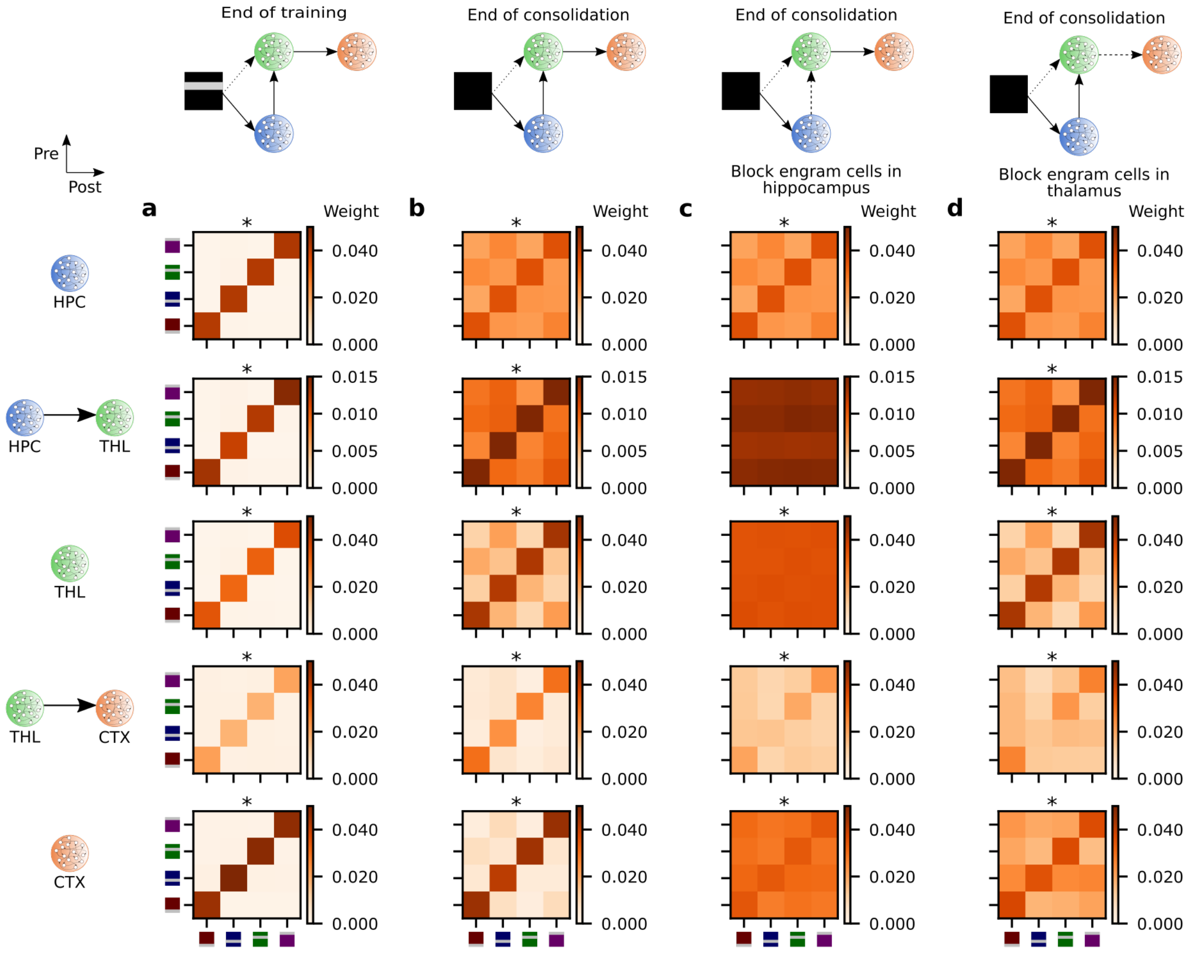

Supplementary Figure 19. Subcortical engram cells are essential for the consolidation of thalamocortical coupling. a-d, Mean weight strength of excitatory synapses onto excitatory neurons clustered according to engram cell preference for the network in Fig. 2a. From top to bottom: HPC (recurrent), HPC→THL (feedforward), THL (recurrent), THL→CTX (feedforward), and CTX (recurrent). Two-sided Kolmogorov-Smirnov test between the distribution of excitatory weights among engram cells encoding the same stimulus (i.e., diagonal) and that of excitatory weights among engram cells encoding different stimuli (i.e., off-diagonal). **a**, Mean weight matrices at the end of the training phase (Fig. 2c-e). HPC: p-value = 0. HPC→THL: p-value = 0. THL: p-value = 0. THL→CTX: p-value = 0. CTX: p-value = 0. **b**, Mean weight matrices after 24 hours of consolidation for the intact (control) network (Fig. 2c). HPC: p-value = 0. HPC→THL: p-value = $6.139623 \cdot 10^{-195}$. THL: p-value = 0. THL→CTX: p-value = 0. CTX: p-value = 0. **c**, Mean weight matrices after 24 hours of consolidation for the network with blocked HPC engram cells during consolidation (Fig. 2d). HPC: p-value = 0. HPC→THL: p-value = 1. THL: p-value = $2.581306 \cdot 10^{-3}$. THL→CTX: p-value = 0. CTX: p-value = $9.302215 \cdot 10^{-135}$. **d**, Mean weight matrices after 24 hours of consolidation for the network with blocked THL engram cells during consolidation (Fig. 2e). HPC: p-value = 0. HPC→THL: p-value = $6.139623 \cdot 10^{-195}$. THL: p-value = 0. THL→CTX: p-value = 0. CTX: p-value = 0. *p-value < 0.05 (see Methods).

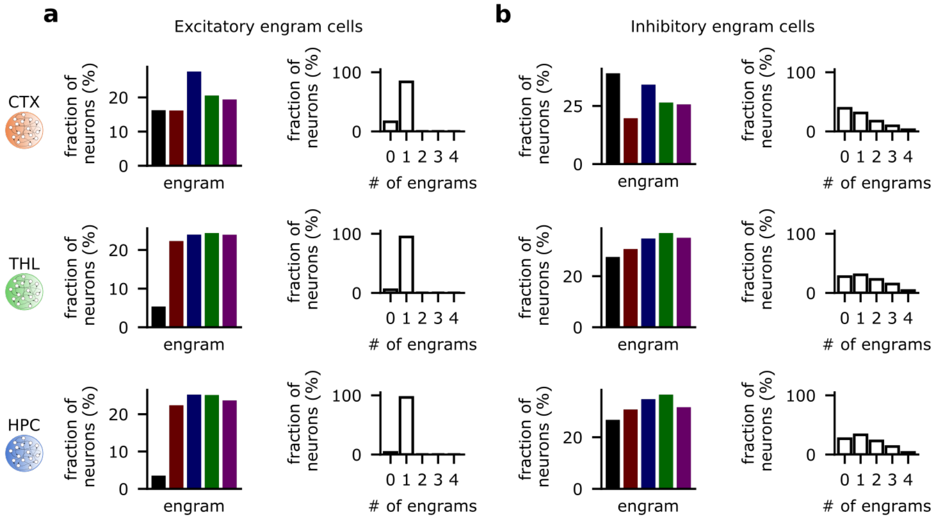

Supplementary Figure 20. Different composition of excitatory and inhibitory engram cells. Analysis of the composition of excitatory and inhibitory engram cells in Fig. 2b. **a**, From left to right: proportion of excitatory neurons that encode each of the stimuli (black denotes no stimulus preference, other colors as in Fig. 2a), and proportion of excitatory neurons that encode 0-4 stimuli. From top to bottom: CTX, THL, and HPC. **b**, Same as **a** but for inhibitory neurons. Near-zero overlap in excitatory engram cells is a consequence of the zero overlap among training stimuli (Fig. 2a).

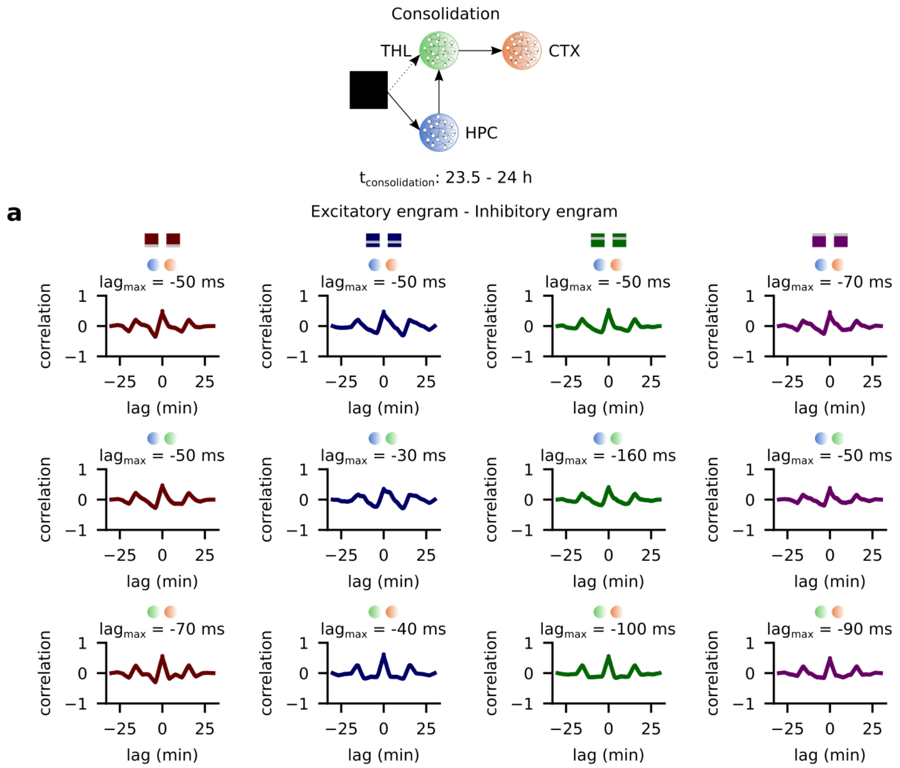

Supplementary Figure 21. Coupled reactivations of excitatory and inhibitory engrams across regions. a, Cross-correlograms of the population activity of excitatory engram cells in one region and the population activity of inhibitory engrams cells in another region during the consolidation phase of Fig. 2c (see Methods for the definition of lag_{max}). Pair of stimuli indicated at the top. Pair of regions indicated at the top of each cross-correlogram: correlation computed between the activity of excitatory engram cells in the region on the left and the activity of inhibitory engram cells in the region on the right. Time interval: between 23.5 and 24 h of consolidation. Color and stimuli as in Fig. 2a.

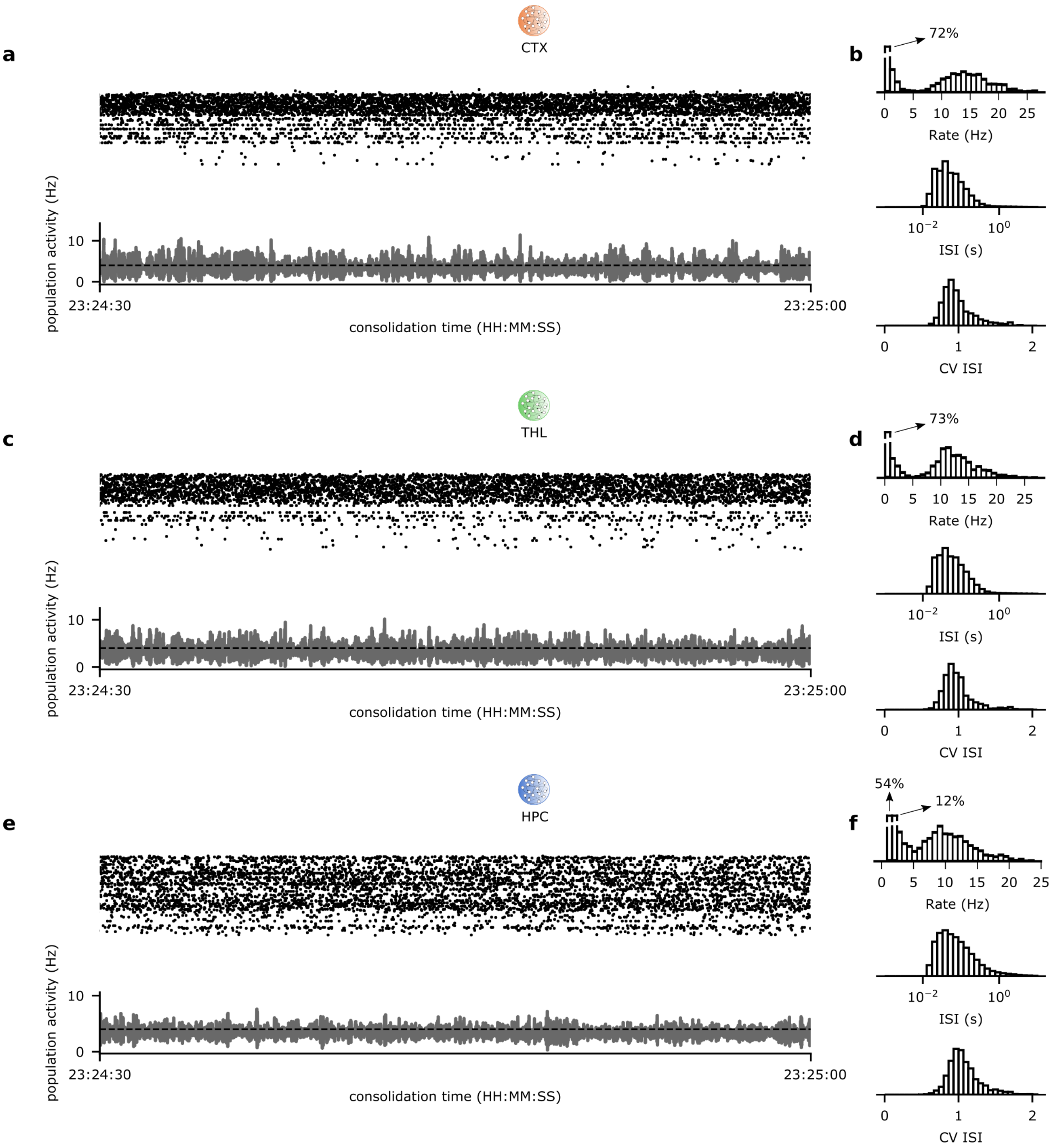

Supplementary Figure 22. Inhibitory plasticity controls network activity in CTX, THL, and HPC. Analysis of excitatory network activity in Fig. 2b. **a**, Spike raster of 256 randomly-chosen excitatory neurons (top) and population activity of all excitatory neurons (bottom) in CTX in a 30-second interval in the consolidation phase. For clarity, only every fifth spike is plotted in the raster. Dashed line in the activity plot indicates target activity level $\gamma = 4$ Hz. Sample neurons with a higher firing rate in the raster plot are part of the engram reactivated in the time interval shown. **b**, Network statistics for **a**. From top to bottom: histograms of firing rates, interspike intervals, and coefficient of variation of interspike intervals (CV ISI). **c**, Same as **a** but for THL. **d**, Same as **b** but for **c**. **e**, Same as **a** but for HPC. **f**, Same as **b** for but for **e**.

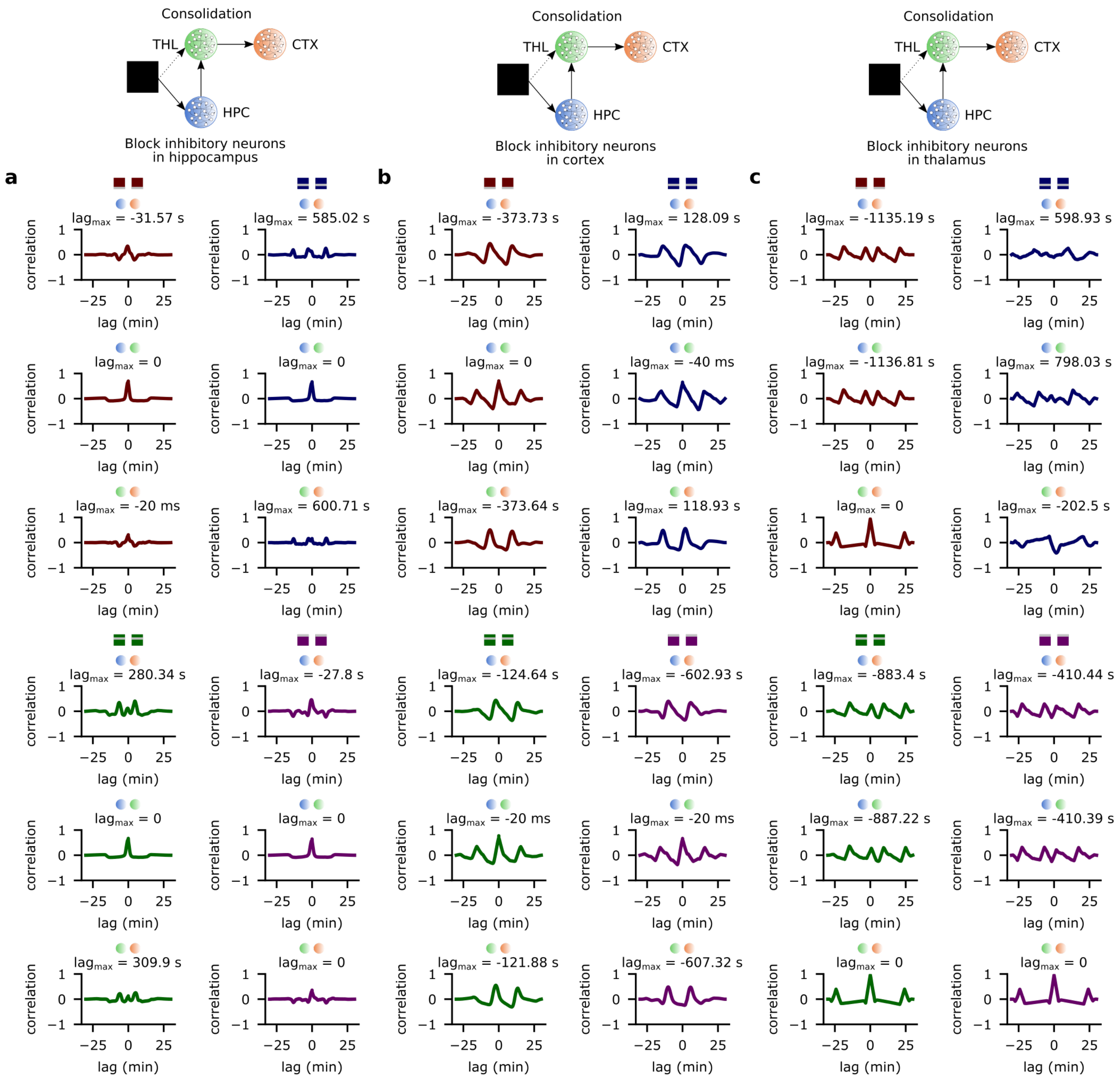

Supplementary Figure 23. Blocking inhibitory neurons decouples engram reactivations in the HPC→THL→CTX circuit. a, Cross-correlograms of the population activities of excitatory engram cells in two different regions during the consolidation phase of Fig. 4a (see Methods for the definition of lag_{max}). Pair of stimuli indicated at the top. Pair of regions indicated at the top of each cross-correlogram. Time interval: between 23.5 and 24 h of consolidation. **b**, Same as **a** but for Fig. 4b. **c**, Same as **a** but for Fig. 4c. **a-c**, Color and stimuli as in Fig. 2a.

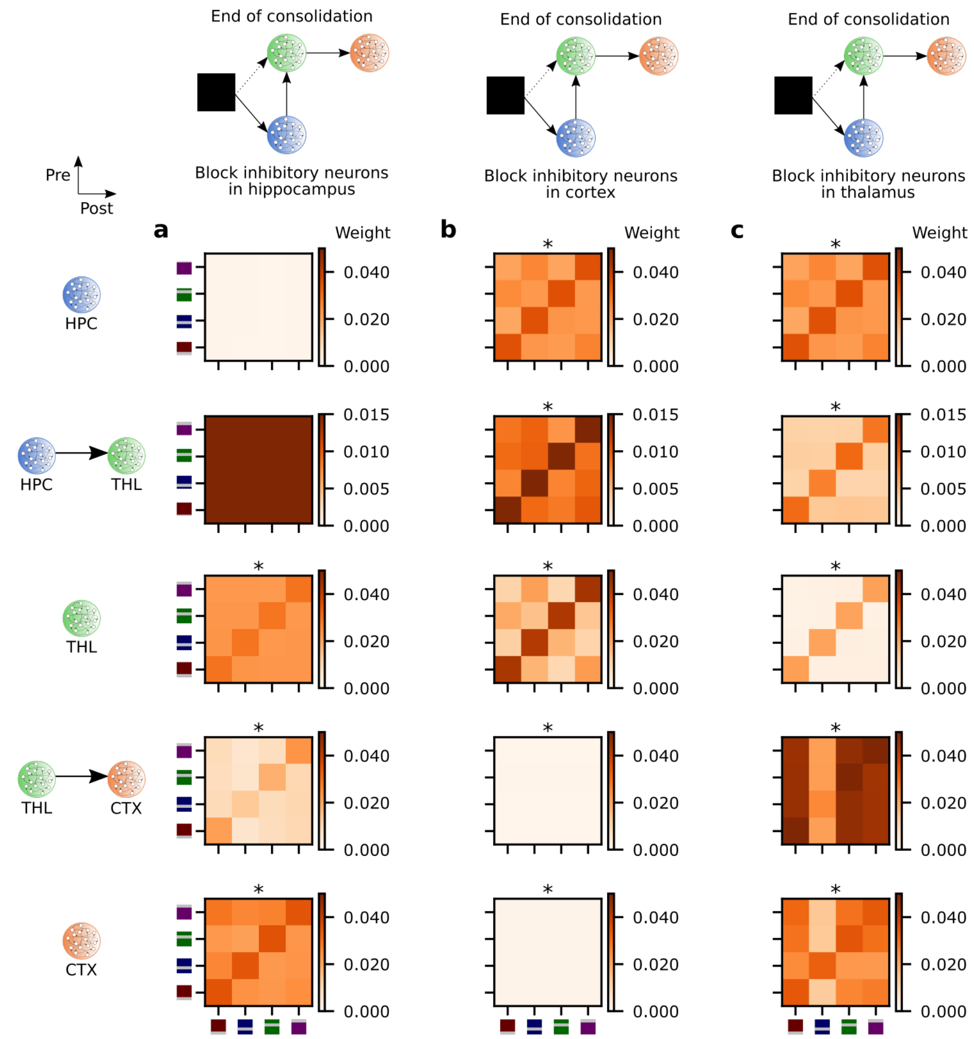

Supplementary Figure 24. Inhibitory neurons are crucial for the consolidation of subcortical-cortical synaptic coupling. Analysis of synaptic coupling in Fig. 4. **a-c**, Mean weight strength of excitatory synapses onto excitatory neurons clustered according to engram cell preference after 24 hours of consolidation. From top to bottom: HPC (recurrent), HPC→THL (feedforward), THL (recurrent), THL→CTX (feedforward), and CTX (recurrent). Two-sided Kolmogorov-Smirnov test between the distribution of excitatory weights among engram cells encoding the same stimulus (i.e., diagonal) and that of excitatory weights among engram cells encoding different stimuli (i.e., off-diagonal). **a**, Mean weight matrices for the network in Fig. 4a. HPC: p-value = 0.993957. HPC→THL: p-value = 1. THL: p-value = 0. THL→CTX: p-value = 0. CTX: p-value = 0. **b**, Mean weight matrices for the network in Fig. 4b. HPC: p-value = 0. HPC→THL: p-value = $6.139623 \cdot 10^{-195}$. THL: p-value = 0. THL→CTX: p-value = $7.573933 \cdot 10^{-75}$. CTX: p-value = $1.146408 \cdot 10^{-10}$. **c**, Mean weight matrices for the network in Fig. 4c. HPC: p-value = 0. HPC→THL: p-value = $2.442765 \cdot 10^{-284}$. THL: p-value = 0. THL→CTX: p-value = $2.151351 \cdot 10^{-138}$. CTX: p-value = 0. *p-value < 0.05 (see Methods). **a-c**, Stimuli as in Fig. 2a.

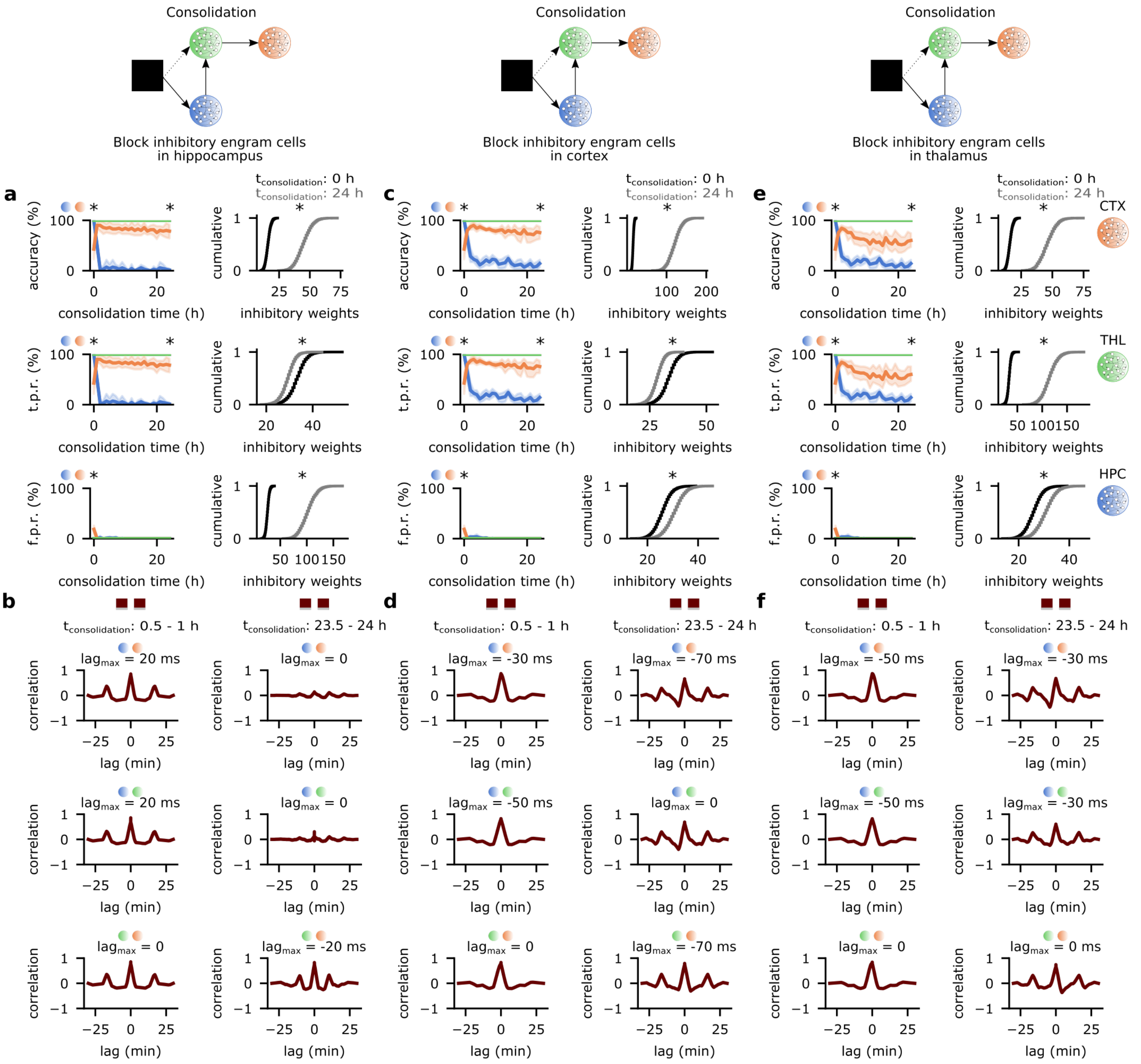

Supplementary Figure 25. Blocking inhibitory engram cells does not prevent CTX engram maturation. a, Left: Memory recall as a function of consolidation time when inhibitory engram cells in HPC are blocked during consolidation in the protocol of Fig. 2b. Top to bottom: accuracy, true positive rate (t.p.r.), and false positive rate (f.p.r.). For each recall metric, two-sided Mann-Whitney U test (MT) between HPC and CTX. Accuracy / t.p.r. (same value for both metrics in each case): consolidation time = 0 (p-value = $7.494958 \cdot 10^{-3}$) and 24 h (p-value = $9.467354 \cdot 10^{-3}$). f.p.r.: consolidation time = 0 (p-value = $7.290358 \cdot 10^{-3}$) and 24 h (p-value = 0.920340). Right: Cumulative distribution of the total inhibitory synaptic weights onto individual excitatory engram cells at consolidation time = 0 (black) and 24 hours (gray). Top to bottom: CTX, THL, and HPC. Two-sided Kolmogorov-Smirnov test (KT) between each pair of weight distributions (CTX/THL/HPC: p-value = 0). **b**, Left/Right: For two consolidation intervals in **a**, cross-correlograms of the population activities of excitatory engram cells encoding the same stimulus in two different regions. Pair of regions indicated at the top of each cross-correlogram (see Methods for the definition of lag_{max}). **c-d**, Same as **a-b** but when inhibitory engram cells in CTX are blocked during consolidation. For each recall metric, MT between HPC and CTX. Accuracy / t.p.r. (same value for both metrics in each case): consolidation time = 0 (p-value = $7.494958 \cdot 10^{-3}$) and 24 h (p-value = $1.192523 \cdot 10^{-2}$). f.p.r.: consolidation time = 0 (p-value = $7.290358 \cdot 10^{-3}$) and 24 h (p-value = 0.920340). KT between each pair of weight distributions (CTX/THL/HPC: p-value = 0). **e-f**, Same as **a-b** but when inhibitory engram cells in THL are blocked during consolidation. For each recall metric, MT between HPC and CTX. Accuracy / t.p.r. (same value for both metrics in each case): consolidation time = 0 (p-value = $7.494958 \cdot 10^{-3}$) and 24 h (p-value = $1.192523 \cdot 10^{-2}$). f.p.r.: consolidation time = 0 (p-value = $7.290358 \cdot 10^{-3}$) and 24 h (p-value = 0.920340). KT between each pair of weight distributions (CTX/THL/HPC: p-value = 0). **a/c/e**, $n = 5$ trials. Mean values and 90% confidence intervals shown. **a-f**, Color and stimuli as in Fig. 2a. *p-value < 0.05 (see Methods).

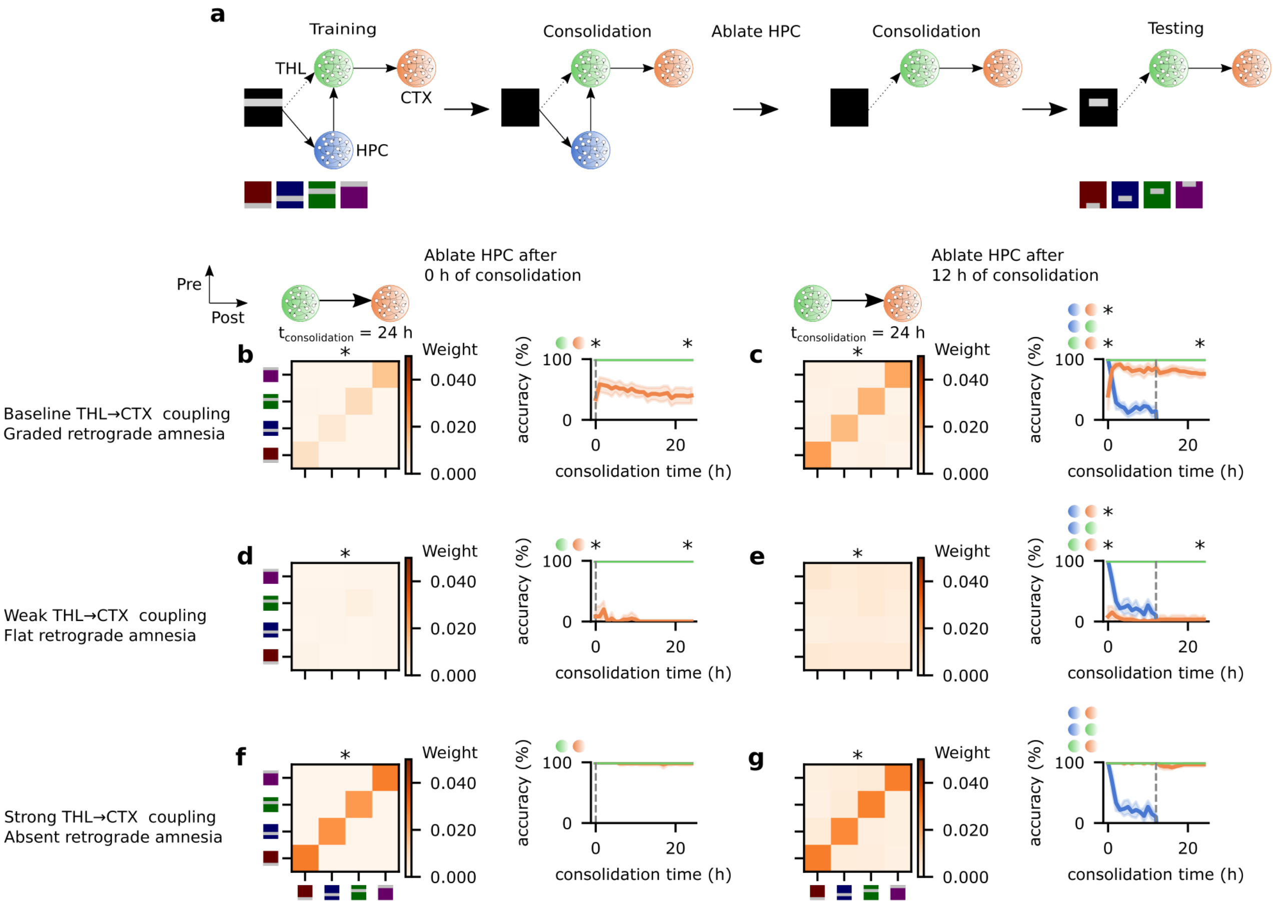

Supplementary Figure 26. THL→CTX coupling shapes retrograde amnesia pattern induced by HPC ablation. **a**, Schematic of simulation protocol with ablation of HPC during the consolidation phase. **b**, Analysis of synaptic coupling and memory recall when HPC is ablated after 0 h of consolidation in protocol **a** (i.e., at the end of the training phase) for the network with $\lambda^{\beta}_{thl\rightarrow ctx} = 105$ (Fig. 2c). Left: Mean weight strength of THL→CTX synapses clustered according to engram cell preference at the end of 24 h of consolidation. Two-sided Kolmogorov-Smirnov test (KT) between the distribution of THL→CTX weights among engram cells encoding the same stimulus (i.e., diagonal) and that of THL→CTX weights among engram cells encoding different stimuli (i.e., off-diagonal): p-value = 0. Right: Memory recall accuracy in the testing phase as a function of consolidation time (dashed vertical line indicates when HPC was ablated). Two-sided Mann-Whitney U test (MT) between accuracy in THL and CTX at consolidation time = 0 (p-value $= 7.088721 \cdot 10^{-3}$) and 24 h (p-value $= 7.494958 \cdot 10^{-3}$). **c**, Same as **b** but when HPC is ablated only after 12 h of consolidation. KT between the distribution of THL→CTX weights among engram cells encoding the same stimulus (i.e., diagonal) and that of THL→CTX weights among engram cells encoding different stimuli (i.e., off-diagonal): p-value = 0. MT between accuracy in pairs of regions at consolidation time = 0 (HPC vs. CTX p-value $= 7.290358 \cdot 10^{-3}$; HPC vs. THL p-value = 0.920340; THL vs. CTX p-value $= 7.290358 \cdot 10^{-3}$) and 24 h (THL vs. CTX p-value $= 7.088721 \cdot 10^{-3}$). **d-e**, Same as **b-c** but for the network with $\lambda^{\beta}_{thl\rightarrow ctx} = 160$ (Fig. 5c). **d**: KT between the distribution of THL→CTX weights among engram cells encoding the same stimulus (i.e., diagonal) and that of THL→CTX weights among engram cells encoding different stimuli (i.e., off-diagonal) (p-value $= 2.953952 \cdot 10^{-13}$); MT between accuracy in THL and CTX at consolidation time = 0 (p-value $= 5.583617 \cdot 10^{-3}$) and 24 h (p-value $= 3.976752 \cdot 10^{-3}$). **e**: KT between the distribution of THL→CTX weights among engram cells encoding the same stimulus (i.e., diagonal) and that of THL→CTX weights among engram cells encoding different stimuli (i.e., off-diagonal) (p-value $= 6.512631 \cdot 10^{-23}$); MT between accuracy in pairs of regions at consolidation time = 0 (HPC vs. CTX p-value $= 5.583617 \cdot 10^{-3}$; HPC vs. THL p-value = 0.920340; THL vs. CTX p-value $= 5.583617 \cdot 10^{-3}$) and 24 h (THL vs. CTX p-value $= 5.583617 \cdot 10^{-3}$). **f-g**, Same as **b-c** but for the network with $\lambda^{\beta}_{thl\rightarrow ctx} = 50$ (Fig. 5g). **f**: KT between the distribution of THL→CTX weights among engram cells encoding the same stimulus (i.e., diagonal) and that of THL→CTX weights among engram cells encoding different stimuli (i.e., off-diagonal) (p-value = 0); MT between accuracy in THL and CTX at consolidation time = 0 (p-value = 0.920340) and 24 h (p-value = 0.423711). **g**: KT between the distribution of THL→CTX weights among engram cells encoding the same stimulus (i.e., diagonal) and that of THL→CTX weights among engram cells encoding different stimuli (i.e., off-diagonal) (p-value = 0); MT between accuracy in pairs of regions at consolidation time = 0 (HPC vs. CTX p-value = 0.920340; HPC vs. THL p-value = 0.920340; THL vs. CTX p-value = 0.920340) and 24 h (THL vs. CTX p-value = 0.177016). *p-value < 0.05 (see Methods). **b-g**, $n = 5$ trials. Mean values and 90% confidence intervals shown. **a-g**, Color and stimuli as in Fig. 2a.

Supplementary Table 1. Summary of network simulation parameters. Value source indicated as below:
(a) for values taken from: Friedemann Zenke, Everton J Agnes, and Wulfram Gerstner. Diverse synaptic plasticity mechanisms orchestrated to form and retrieve memories in spiking neural networks. *Nature Communications*, 6(1):1–13, 2015.
(b) for values chosen somewhat arbitrarily without targeted optimization.
(c) for values optimized over several preliminary simulations.

**Neural Populations**			
**Parameter**	**Figure**	**Value[source]**	**Description**
N_{exc}	All	4096[a]	Number of excitatory neurons in each model region
N_{inh}	All	1024[a]	Number of inhibitory neurons in each model region
N_{stim}	All	4096[a]	Number of stimulus neurons
N_{ext}^{ctx}	2,5,Supp. 17	4096[b]	Number of external background neurons projecting to CTX
N_{ext}^{hpc}	2,5,Supp. 17	4096[b]	Number of external background neurons projecting to HPC
**Network Connectivity**			
**Parameter**	**Figure**	**Value[source]**	**Description**
ϵ_{rec}	All	0.05[a]	Probability of connection of recurrent synapses (EE, EI, II, IE)
ϵ_{stim}	1,Supp. 6	0.1[c]	Probability of connection of STIM→CTX and STIM→HPC
$\epsilon_{hpc\to ctx}$	Supp. 6	0.02[c]	Probability of connection of HPC→CTX
R_{hpc}	2,5,Supp. 17	8[c]	Radius of receptive field in STIM→HPC
R_{thl}	2,5,Supp. 17	4[c]	Radius of receptive field in STIM→THL
$\epsilon_{hpc\to thl}$	2,5,Supp. 17	0.02[c]	Probability of connection of HPC→THL
$\epsilon_{thl\to ctx}$	2,5,Supp. 17	0.05[c]	Probability of connection of THL→CTX
$\epsilon_{ext\to ctx}$	2,5,Supp. 17	0.1[c]	Probability of connection of external neurons to CTX
$\epsilon_{ext\to hpc}$	2,5,Supp. 17	0.1[c]	Probability of connection of external neurons to HPC
$\epsilon_{ctx\to rdt}$	Supp. 16,Supp. 17	0.05[c]	Probability of connection of CTX→RDT synapses
$\epsilon_{hpc\to rdt}$	Supp. 16,Supp. 17	0.05[c]	Probability of connection of HPC→RDT synapses
$\epsilon_{thl\to rdt}$	Supp. 17	0.05[c]	Probability of connection of THL→RDT synapses
w^{EE}	All	0.1[a]	Initial weight of recurrent EE synapses
w^{EI}	All	0.6[a]	Fixed weight of recurrent EI synapses
w^{II}	All	0.2[a]	Fixed weight of recurrent II synapses
w^{IE}	All	0.2[a]	Initial weight of recurrent IE synapses
w_{stim}	1,Supp. 6	0.1[c]	Initial weight of STIM→CTX and STIM→HPC synapses
$w_{hpc\to ctx}$	Supp. 6	0.1[c]	Initial weight of HPC→CTX synapses
$w_{stim\to hpc}$	2,5,Supp. 17	0.5[c]	Initial weight of STIM→HPC synapses
$w_{stim\to thl}$	2,5,Supp. 17	2.0[c]	Fixed weight of STIM→THL synapses
$w_{hpc\to thl}$	2,5,Supp. 17	0.1[c]	Initial weight of HPC→THL synapses
$w_{thl\to ctx}$	2,5	0.15[c]	Initial weight of THL→CTX synapses
	Supp. 17	0.2[c]	
$w_{ext\to ctx}$	2,5,Supp. 17	0.2[b]	Fixed weight of synapses from external neurons to CTX
$w_{ext\to hpc}$	2,5,Supp. 17	0.2[b]	Fixed weight of synapses from external neurons to HPC
$w_{ctx\to rdt}$	Supp. 16,Supp. 17	0.05[c]	Initial weight of CTX→RDT synapses
$w_{hpc\to rdt}$	Supp. 16,Supp. 17	0.05[c]	Initial weight of HPC→RDT synapses
$w_{thl\to rdt}$	Supp. 17	0.05[c]	Initial weight of THL→RDT synapses
**Neuron Model**			
**Parameter**	**Figure**	**Value[source]**	**Description**
τ^{m}	All	20 ms[a]	Membrane time constant
U^{rest}	All	-70 mV[a]	Membrane resting potential
U^{exc}	All	0 mV[a]	Excitatory reversal potential
U^{inh}	All	-80 mV[a]	Inhibitory reversal potential
τ^{thr}	All	5 ms[a]	Threshold time constant
ϑ^{rest}	All	-50 mV[a]	Threshold resting value
ϑ^{spike}	All	100 mV[a]	Threshold value immediately after spike

**Synapse Model**			
**Parameter**	**Figure**	**Value**[source]	**Description**
τ^{gaba}	All	10 ms[a]	GABA decay time constant
τ^{a}	All	100 ms[a]	Adaptation time constant
Δ^{a}	All	0.1[a]	Adaptation strength
α^{E}	All	0.2[a]	AMPA/NMDA ratio for excitatory neurons
α^{I}	All	0.3[a]	AMPA/NMDA ratio for inhibitory neurons
τ^{ampa}	All	5 ms[a]	AMPA decay time constant
τ^{nmda}	All	100 ms[a]	NMDA decay time constant
**Short-Term Plasticity Model**			
**Parameter**	**Figure**	**Value**[source]	**Description**
τ^{d}_{EE}	All	150 ms[a]	Depression time constant for EE synapses
τ^{d}_{EI}	All	200 ms[a]	Depression time constant for EI synapses
τ^{f}	All	600 ms[a]	Facilitation time constant for excitatory synapses
U	All	0.2[a]	Initial release probability for excitatory synapses
**Long-Term Excitatory Synaptic Plasticity Model**			
**CTX EE**			
**Parameter**	**Figure**	**Value**[source]	**Description**
η^{exc}_{ctx}	All	1 x 10^{-3}[c]	Learning rate of EE synapses in CTX
λ^{β}_{ctx}	All	50[c]	$\beta_{ctx}/\eta^{exc}_{ctx}$ ratio for EE synapses in CTX
τ^{cons}_{ctx}	All	20 min[c]	Synaptic consolidation time constant for EE synapses in CTX
**HPC EE**			
**Parameter**	**Figure**	**Value**[source]	**Description**
η^{exc}_{hpc}	1,Supp. 6	1.25 x 10^{-3}[c]	Learning rate of EE synapses in HPC
	2,5,Supp. 17	1.5 x 10^{-3}[c]	
λ^{β}_{hpc}	1,Supp. 6	25[c]	$\beta_{hpc}/\eta^{exc}_{hpc}$ ratio for EE synapses in HPC
	2,5,Supp. 17	50[c]	
τ^{cons}_{hpc}	1,Supp. 6	3 h[c]	Synaptic consolidation time constant for EE synapses in HPC
	2,5,Supp. 17	20 min[c]	
**STIM→CTX**			
**Parameter**	**Figure**	**Value**[source]	**Description**
$\eta^{exc}_{stim\rightarrow ctx}$	1,Supp. 6	1 x 10^{-3}[c]	Learning rate of STIM→CTX synapses
$\lambda^{\beta}_{stim\rightarrow ctx}$	1,Supp. 6	85[c]	$\beta_{stim\rightarrow ctx}/\eta^{exc}_{stim\rightarrow ctx}$ ratio for STIM→CTX synapses
$\tau^{cons}_{stim\rightarrow ctx}$	1,Supp. 6	20 min[c]	Synaptic consolidation time constant for STIM→CTX synapses
**STIM→HPC**			
**Parameter**	**Figure**	**Value**[source]	**Description**
$\eta^{exc}_{stim\rightarrow hpc}$	1,Supp. 6	1.25 x 10^{-3}[c]	Learning rate of STIM→HPC synapses
	2,5,Supp. 17	1.5 x 10^{-3}[c]	
$\lambda^{\beta}_{stim\rightarrow hpc}$	1,Supp. 6	35[c]	$\beta_{stim\rightarrow hpc}/\eta^{exc}_{stim\rightarrow hpc}$ ratio for STIM→HPC synapses
	2,5,Supp. 17	30[c]	
$\tau^{cons}_{stim\rightarrow hpc}$	All	3 h[c]	Synaptic consolidation time constant for STIM→HPC synapses
**HPC→CTX**			
**Parameter**	**Figure**	**Value**[source]	**Description**
$\eta^{exc}_{hpc\rightarrow ctx}$	Supp. 6	1 x 10^{-3}[c]	Learning rate of HPC→CTX synapses
$\lambda^{\beta}_{hpc\rightarrow ctx}$	Supp. 6A-F	85[c]	$\beta_{hpc\rightarrow ctx}/\eta^{exc}_{hpc\rightarrow ctx}$ ratio for HPC→CTX synapses
	Supp. 6G-I	Supp. 170[c]	
$\tau^{cons}_{hpc\rightarrow ctx}$	Supp. 6	20 min[c]	Synaptic consolidation time constant for HPC→CTX synapses
**THL EE**			
**Parameter**	**Figure**	**Value**[source]	**Description**
η^{exc}_{thl}	2,5,Supp. 17	1.5 x 10^{-3}[c]	Learning rate of EE synapses in THL
λ^{β}_{thl}	2,5,Supp. 17	50[c]	$\beta_{thl}/\eta^{exc}_{thl}$ ratio for EE synapses in THL
τ^{cons}_{thl}	2,5,Supp. 17	20 min[c]	Synaptic consolidation time constant for EE synapses in THL

**HPC→THL**			
**Parameter**	**Figure**	**Value[source]**	**Description**
$\eta^{exc}_{hpc\rightarrow thl}$	2,5,Supp. 17	1.5 x 10^{-3}[c]	Learning rate of HPC→THL synapses
$\lambda^{\beta}_{hpc\rightarrow thl}$	2,5,Supp. 17	50[c]	$\beta_{hpc\rightarrow thl}/\eta^{exc}_{hpc\rightarrow thl}$ ratio for HPC→THL synapses
$\tau^{cons}_{hpc\rightarrow thl}$	2,5,Supp. 17	20 min[c]	Synaptic consolidation time constant for HPC→THL synapses
**THL→CTX**			
**Parameter**	**Figure**	**Value[source]**	**Description**
$\eta^{exc}_{thl\rightarrow ctx}$	2,5,Supp. 17	1 x 10^{-3}[c]	Learning rate of THL→CTX synapses
$\lambda^{\beta}_{thl\rightarrow ctx}$	2,5B,Supp. 17	105[c]	$\beta_{thl\rightarrow ctx}/\eta^{exc}_{thl\rightarrow ctx}$ ratio for THL→CTX synapses
	5C-F	160[c]	
	5G-J	50[c]	
$\tau^{cons}_{thl\rightarrow ctx}$	2,5,Supp. 17	20 min[c]	Synaptic consolidation time constant for THL→CTX synapses
**RDT EE**			
**Parameter**	**Figure**	**Value[source]**	**Description**
η^{exc}_{rdt}	Supp. 16	1.25 x 10^{-3}[c]	Learning rate of EE synapses in RDT
	Supp. 17	1.5 x 10^{-3}[c]	
λ^{β}_{rdt}	Supp. 16,Supp. 17	50[c]	$\beta_{rdt}/\eta^{exc}_{rdt}$ ratio for EE synapses in RDT
τ^{cons}_{rdt}	Supp. 16,Supp. 17	20 min[c]	Synaptic consolidation time constant for EE synapses in RDT
**CTX→RDT**			
**Parameter**	**Figure**	**Value[source]**	**Description**
$\eta^{exc}_{ctx\rightarrow rdt}$	Supp. 16	1.25 x 10^{-3}[c]	Learning rate of CTX→RDT synapses
	Supp. 17	1.5 x 10^{-3}[c]	
$\lambda^{\beta}_{ctx\rightarrow rdt}$	Supp. 16	85[c]	$\beta_{ctx\rightarrow rdt}/\eta^{exc}_{ctx\rightarrow rdt}$ ratio for CTX→RDT synapses
	Supp. 17	50[c]	
$\tau^{cons}_{ctx\rightarrow rdt}$	Supp. 16,Supp. 17	20 min[c]	Synaptic consolidation time constant for CTX→RDT synapses
**HPC→RDT**			
**Parameter**	**Figure**	**Value[source]**	**Description**
$\eta^{exc}_{hpc\rightarrow rdt}$	Supp. 16	1.25 x 10^{-3}[c]	Learning rate of HPC→RDT synapses
	Supp. 17	1.5 x 10^{-3}[c]	
$\lambda^{\beta}_{hpc\rightarrow rdt}$	Supp. 16	50[c]	$\beta_{hpc\rightarrow rdt}/\eta^{exc}_{hpc\rightarrow rdt}$ ratio for HPC→RDT synapses
	Supp. 17	30[c]	
$\tau^{cons}_{hpc\rightarrow rdt}$	Supp. 16	20 min[c]	Synaptic consolidation time constant for HPC→RDT synapses
	Supp. 17	3 h[c]	
**THL→RDT**			
**Parameter**	**Figure**	**Value[source]**	**Description**
$\eta^{exc}_{thl\rightarrow rdt}$	Supp. 17	1.5 x 10^{-3}[c]	Learning rate of THL→RDT synapses
$\lambda^{\beta}_{thl\rightarrow rdt}$	Supp. 17	50[c]	$\beta_{thl\rightarrow rdt}/\eta^{exc}_{thl\rightarrow rdt}$ ratio for THL→RDT synapses
$\tau^{cons}_{thl\rightarrow rdt}$	Supp. 17	20 min[c]	Synaptic consolidation time constant for THL→RDT synapses
**All Excitatory Synapses with Long-Term Plasticity**			
**Parameter**	**Figure**	**Value[source]**	**Description**
λ^{δ}	All	0.02[c]	δ/η^{exc} ratio
A	All	1[a]	LTP rate
τ^{+}	All	20 ms[a]	Time constant of presynaptic trace
τ^{-}	All	20 ms[a]	Time constant of postsynaptic trace
τ^{slow}	All	100 ms[a]	Time constant of slow postsynaptic trace
P	All	20[a]	Potential strength
w^{P}	All	0.5[a]	Upper fixed point of reference weight potential
$\tilde{w}$	All	0.0[a]	Initial reference weight
τ^{hom}	All	10 min[a]	Time constant of homeostatic regulation
τ^{ht}	All	100 ms[a]	Time constant of postsynaptic trace for homeostatic regulation
w^{max}_{exc}	All	5.0[a]	Maximum excitatory synaptic weight
w^{min}_{exc}	All	0.0[a]	Minimum excitatory synaptic weight

**Inhibitory Synaptic Plasticity Model**			
**Parameter**	**Figure**	**Value(source)**	**Description**
λ^{η}	All	50[c]	η^{exc}/η^{inh} ratio
γ	All	4 Hz[a]	Target activity level for each model region
τ^{iSTDP}	All	20 ms[a]	Time constant of pre- and postsynaptic traces
τ^{H}	All	10 s[a]	Time constant of global secreted factor
w_{inh}^{max}	All	5.0[a]	Maximum inhibitory synaptic weight
w_{inh}^{min}	All	0.0[a]	Minimum inhibitory synaptic weight
**Stimulus Model**			
**Parameter**	**Figure**	**Value(source)**	**Description**
ν^{bg}	All	5 Hz[a]	Firing rate of stimulus population except during consolidation
ν^{con}	1,Supp. 6,Supp. 16	2 Hz[c]	Firing rate of stimulus population in the consolidation phase
	2,5,Supp. 17	0 Hz[c]	
ν^{stim}	All	25 Hz[a]	Firing rate of neurons in a given stimulus when it is presented
$T_{On}^{training}$	All	1 s[a]	Mean stimulus-on period in the training phase
$T_{Off}^{training}$	All	2 s[a]	Mean stimulus-off period in the training phase
$T_{On}^{testing}$	1,Supp. 6,Supp. 16	1 s[b]	Mean stimulus-on period in the testing phase
	2,5,Supp. 17	2 s[b]	
$T_{Off}^{testing}$	All	2 s[b]	Mean stimulus-off period in the testing phase
**External Populations Model**			
**Parameter**	**Figure**	**Value(source)**	**Description**
ν_{ext}^{bg}	2,5,Supp. 17	0 Hz[b]	Firing rate of external neurons except in the consolidation phase
ν_{ext}^{con}	2,5,Supp. 17	1 Hz[b]	Firing rate of external neurons in the consolidation phase
**Network Simulation**			
**Parameter**	**Figure**	**Value(source)**	**Description**
T_{burn}	All	120 s[a]	Duration of burn-in period prior to training
$T_{training}$	1,Supp. 6,Supp. 16	45 min[c]	Duration of training phase
	2,5	30 min[c]	
	Supp. 17	25 min[c]	
$T_{consolidation}$	1,Supp. 6,Supp. 16	12 h[b]	Duration of consolidation phase
	2,5,Supp. 17	24 h[b]	
$T_{testing}$	All	60 s[b]	Duration of testing phase
Δ	All	0.1 ms[a]	Time step for updating neuronal state variables except for $\tilde{w}$
Δ_{long}	All	1.2 s[a]	Time step for updating $\tilde{w}$
N_{ranks}	All	16[c]	Number of MPI ranks in Auryn simulations
