# Supplementary material for: Coordinated hippocampal-thalamic-cortical communication crucial for engram dynamics underneath systems consolidation
Source: Nat Commun. 2022 Feb 11;13:840. doi: 10.1038/s41467-022-28339-z (PMC8837777; doi:10.1038/s41467-022-28339-z)
Supplement: Supplementary file 3 — Reporting Summary [file 41467_2022_28339_MOESM3_ESM.pdf]

nature research

Corresponding author(s): Claudia Clopath

Last updated by author(s): Dec 13, 2021

Reporting Summary

Nature Research wishes to improve the reproducibility of the work that we publish. This form provides structure for consistency and transparency in reporting. For further information on Nature Research policies, see our Editorial Policies and the Editorial Policy Checklist.

Statistics

For all statistical analyses, confirm that the following items are present in the figure legend, table legend, main text, or Methods section.

n/a	Confirmed	
☐	☒	The exact sample size (n) for each experimental group/condition, given as a discrete number and unit of measurement
☒	☐	A statement on whether measurements were taken from distinct samples or whether the same sample was measured repeatedly
☐	☒	The statistical test(s) used AND whether they are one- or two-sided<br>*Only common tests should be described solely by name; describe more complex techniques in the Methods section.*
☒	☐	A description of all covariates tested
☐	☒	A description of any assumptions or corrections, such as tests of normality and adjustment for multiple comparisons
☐	☒	A full description of the statistical parameters including central tendency (e.g. means) or other basic estimates (e.g. regression coefficient) AND variation (e.g. standard deviation) or associated estimates of uncertainty (e.g. confidence intervals)
☐	☒	For null hypothesis testing, the test statistic (e.g. F, t, r) with confidence intervals, effect sizes, degrees of freedom and P value noted<br>*Give P values as exact values whenever suitable.*
☒	☐	For Bayesian analysis, information on the choice of priors and Markov chain Monte Carlo settings
☒	☐	For hierarchical and complex designs, identification of the appropriate level for tests and full reporting of outcomes
☒	☐	Estimates of effect sizes (e.g. Cohen's d, Pearson's r), indicating how they were calculated

Our web collection on statistics for biologists contains articles on many of the points above.

Software and code

Policy information about availability of computer code

Data collection	We wrote C++ code using the Auryn framework for spiking neural network simulation version 6928b97 to perform all reported simulations. The code is available at https://doi.org/10.5281/zenodo.5772014.
Data analysis	We wrote Python 3 (version 3.8 or later) code to analyze the results of all reported simulations using the the following packages: numpy 1.20.1, pandas 1.2.2, cython 0.29.22, scikit-learn 0.24.1, scipy 1.6.1, matplotlib 3.3.4, and seaborn 0.11.1. The code is available at https://doi.org/10.5281/zenodo.5772014.

For manuscripts utilizing custom algorithms or software that are central to the research but not yet described in published literature, software must be made available to editors and reviewers. We strongly encourage code deposition in a community repository (e.g. GitHub). See the Nature Research guidelines for submitting code & software for further information.

Data

Policy information about availability of data

All manuscripts must include a data availability statement. This statement should provide the following information, where applicable:

- Accession codes, unique identifiers, or web links for publicly available datasets
- A list of figures that have associated raw data
- A description of any restrictions on data availability

The data necessary to reproduce the simulations reported in this study are available in a public repository at https://doi.org/10.5281/zenodo.5772014.

Field-specific reporting

Please select the one below that is the best fit for your research. If you are not sure, read the appropriate sections before making your selection.

☒ Life sciences ☐ Behavioural & social sciences ☐ Ecological, evolutionary & environmental sciences

For a reference copy of the document with all sections, see nature.com/documents/nr-reporting-summary-flat.pdf

Life sciences study design

All studies must disclose on these points even when the disclosure is negative.

Sample size	No statistical methods were used to predetermine sample sizes. Sample sizes were selected on the basis of previous studies. The selected sample sizes were sufficient given the reproducibility of our results across trials.
Data exclusions	No data were excluded from the analyses.
Replication	The simulations reported in the manuscript were performed on three different machines and the main findings of the study were replicated each time.
Randomization	All spiking recurrent neural networks trained in the study were randomly initialized (with random seeds) before training.
Blinding	Blinding was not performed for the analysis of simulation results since simulation output was automatically processed by our custom data analysis code without human intervention.

Reporting for specific materials, systems and methods

We require information from authors about some types of materials, experimental systems and methods used in many studies. Here, indicate whether each material, system or method listed is relevant to your study. If you are not sure if a list item applies to your research, read the appropriate section before selecting a response.

Materials & experimental systems

n/a	Involved in the study	
☒	☐	Antibodies
☒	☐	Eukaryotic cell lines
☒	☐	Palaeontology and archaeology
☒	☐	Animals and other organisms
☒	☐	Human research participants
☒	☐	Clinical data
☒	☐	Dual use research of concern

Methods

n/a	Involved in the study	
☒	☐	ChIP-seq
☒	☐	Flow cytometry
☒	☐	MRI-based neuroimaging
